# Association and interaction of blood homocysteine and p‐tau217 levels with temporal cortical thinning and cognitive impairment in Alzheimer's disease

**DOI:** 10.1002/alz.70465

**Published:** 2025-07-17

**Authors:** Qiang Wang, Xiaomei Zhong, Pengbo Gao, Zhidai Xiao, Ben Chen, Haoye Tan, Qin Liu, Kexin Yao, Shuang Liang, JiaFu Li, Mingfeng Yang, Danyan Xu, Gaohong Lin, Zhangying Wu, Haishan Shi, Min Zhang, Xiaolei Shi, Huanmin Liu, Yijie Zeng, Yunheng Chen, Yicheng Lin, Shuwei Zhang, Cong Ouyang, Shihan Tian, Yujing Gan, Mingyong Zeng, Jieqin Lv, Xiang Liang, Yuwang Cheng, Jianwen Guo, Pengcheng Ran, Yuping Ning, Huarong Zhou

**Affiliations:** ^1^ The Affiliated Brain Hospital, Guangzhou Medical University Guangzhou Guangdong Province China; ^2^ Key Laboratory of Neurogenetics and Channelopathies of Guangdong Province and the Ministry of Education of China Guangzhou Medical University Guangzhou Guangdong Province China; ^3^ Departments of Nuclear Medicine The Second Affiliated Hospital of Guangzhou University of Chinese Medicine Guangzhou Guangdong Province China; ^4^ State Key Laboratory of Dampness Syndrome of Chinese Medicine The Secondary Hospital of Guangzhou University of Chinese Medicine, Guangdong Provincial Hospital of Traditional Chinese Medicine Guangzhou China

**Keywords:** Alzheimer's disease, amyloid beta, blood biomarkers, homocysteine, mild cognitive impairment, p‐tau217

## Abstract

**INTRODUCTION:**

The relationships between blood homocysteine (Hcy), amyloid beta (Aβ), tau pathology, and their combined effects on cortical thinning and cognitive impairment in Alzheimer's disease (AD) remain poorly understood.

**METHODS:**

Participants were stratified into Aβ+ and Aβ− groups by positron emission tomography. Blood levels of Hcy, AD biomarkers (phosphorylated tau‐217 [p‐tau217], p‐tau181, and Aβ), cortical thickness (via magnetic resonance imaging), and cognitive performance were assessed.

**RESULTS:**

Aβ+ individuals exhibited increased blood Hcy and p‐tau217 levels, which negatively correlated with temporal cortical thickness and cognitive function. A significant interaction between Hcy and p‐tau217 was observed in Aβ+ participants, with high Hcy exacerbating the detrimental effects of p‐tau217 on temporal cortical thinning and cognitive deficits. In Aβ− individuals, Hcy levels were independently associated with tau pathology.

**DISCUSSION:**

Increased Hcy and p‐tau217 levels synergistically contribute to cortical thinning and cognitive impairment, highlighting that Hcy may be a modifiable risk factor for AD progression.

**Highlights**

- Increased blood levels of homocysteine (Hcy) and phosphorylated tau‐217 (p‐tau217) are independently and interactively associated with temporal cortical thinning and cognitive deficits.
- Hcy may predominantly affect tau pathology compared to amyloid aggregation, with its impact on neurodegeneration depending on coexisting pathologies.
- Hcy may serve as a supplementary indicator rather than as an independent predictor in Alzheimer's disease.
- Hcy may serve as a modifiable risk factor, whereas p‐tau217 as a superior diagnostic biomarker and potential therapeutic target.

## 1 BACKGROUND

Alzheimer's disease (AD) is a progressive neurodegenerative disorder characterized by amyloid beta (Aβ) plaques, tau neurofibrillary tangles, cortical atrophy (particularly in the temporal cortex and hippocampus), and cognitive impairment.^1^ Traditionally, Aβ pathology has been considered the primary driver of AD, initiating subsequent tau pathology, neuroinflammation, and neurodegeneration.^2^ However, emerging evidence increasingly challenges the central role of Aβ in AD pathogenesis. Clinical trials that target Aβ clearance (e.g., anti‐Aβ monoclonal antibodies) have reported limited efficacy in halting cognitive decline, despite reducing plaque burden.^3^, ^4^ In addition, individuals who are Aβ‐negative (Aβ–) often exhibit neurodegenerative disease‐related phenotypes, including tau pathology, accelerated cortical thinning, and progressive cognitive dysfunction.^5^, ^6^, ^7^ Compared with Aβ pathology, phosphorylated tau (p‐tau) shows a stronger association with cortical thinning and cognitive impairment.^8^ These findings emphasize the importance of investigating other modifiable metabolic cofactors, such as homocysteine (Hcy), which may interact with Aβ and tau to drive the progression of AD.

Hcy, a sulfur‐containing amino acid associated previously with vascular diseases,^9^ has re‐emerged as a crucial mediator of AD pathogenesis.^10^ Early studies have attributed the Hcy‐related risk of dementia to nonspecific vascular injury.^11^ However, recent advancements have illuminated its direct role in the core pathology of AD. (1) Hcy upregulates beta‐site amyloid precursor protein cleaving enzyme 1 (BACE1) to enhance Aβ production and modulate the Aβ–tau axis.^12^ Hcy induces tau hyperphosphorylation via hypomethylation of tau kinase promoters (e.g., Glycogen Synthase Kinase 3 Beta：GSK3β), promoting oxidative stress and inflammation.^9^, ^13^, ^14^ (2) Elevated blood Hcy levels correlate with cognitive impairment,^15^ oxidative stress,^16^ and neuroinflammation.^17^ (3) Higher Hcy level is linked to thinner cortical gray matter.^18^, ^19^ The three of which are associated with neurodegenerative processes.

Recent advances in blood biomarkers, particularly plasma phosphorylated tau‐217 (p‐tau217) levels, have attracted significant attention due to their superior efficacy compared to other biomarkers.^20^, ^21^ Several studies have shown that p‐tau217 levels are independently associated with Aβ‐ and tau‐PET (positron emission tomography) and mediate the relationship between Aβ and tau.^22^, ^23^ In addition, plasma p‐tau217 levels correlate with cerebrospinal fluid (CSF) Aβ and p‐tau levels, as well as the severity of cognitive impairment.^24^, ^25^ Therefore, an increase in these levels reflects tau pathology and may also serve as an indirect marker of Aβ deposition. However, the relationships between p‐tau217 levels, cortical thinning, and cognitive impairment, especially regarding metabolic factors such as Hcy, remain underexplored.

This study examined the synergistic effects of Hcy and p‐tau217 on temporal cortical thinning and cognitive impairment in Aβ‐stratified cohorts. We hypothesize that (1) in Aβ‐positive individuals, Hcy potentiates tau‐mediated neurodegeneration and accelerates cortical thinning and cognitive impairment through Aβ–tau synergism and vascular mechanisms. (2) In Aβ‐negative individuals, Hcy directly promotes cortical thinning and cognitive deficit via tau phosphorylation and vascular mechanisms, independent of Aβ.

Specifically, this study aims to (1) examine the independent and interactive effects of Hcy and p‐tau217 on temporal cortical thinning and cognitive impairment and (2) identify potential differences in these associations between Aβ‐positive and Aβ‐negative participants. By evaluating the roles of Hcy and p‐tau217 in cortical thinning and cognitive function, this study offers a framework for delineating Aβ‐dependent and Aβ‐independent mechanisms of neurodegeneration, providing new insights into the metabolic contributions to AD progression.

## 2 METHODS

### 2.1 Study cohorts and participants

The study recruited 236 voluntary participants from the Southern China Aging Brain Initiative (SCABI) cohort, all of whom underwent an 18F‐florbetapir PET scan between March 2021 and January 2024. The participant enrollment flowchart is illustrated in Figure S1. All participants or their legal guardians provided signed informed consent to participate in the study. The study adhered to the Declaration of Helsinki and was approved by the Affiliated Brain Hospital Ethics Committees of Guangzhou Medical University.

The SCABI cohort has been detailed in previous studies.^26^ It included participants who met the following criteria: (1) ≥50 years of age; and (2) cognitively normal, or mild cognitive impairment (MCI; according to the Peterson criteria^27^), or dementia (according to the criteria for any dementia according to the Diagnostic and Statistical Manual of Mental Disorders, Fourth Edition [DSM‐IV]^28^). The exclusion criteria were: (1) malignant tumors and severe cerebrovascular diseases (including ischemic stroke and intracerebral hemorrhage with neurological deficit symptoms); (2) severe neurological disorders (such as metabolic brain diseases, encephalitis, multiple sclerosis, epilepsy, traumatic brain injury, normal pressure hydrocephalus, etc.); (3) severe mental illness (schizophrenia, bipolar affective disorder, schizoaffective disorder, paranoid psychosis, intellectual disability, etc.); and (4) systemic diseases causing cognitive impairment (liver dysfunction, renal insufficiency, thyroid dysfunction, severe anemia, folate or vitamin B12 deficiency, syphilis, HIV infection, alcohol, drug abuse, etc.). The first three categories were confirmed by clinical neurological examination, whereas the fourth category was based on self‐report from patients or their families, rather than on laboratory testing.

AD was diagnosed according to the criteria for dementia due to AD in the diagnostic guidelines for probable AD developed by the National Institute on Aging–Alzheimer's Association (NIA‐AA) workgroups^29^ and further confirmed via positive Aβ‐PET.

**RESEARCH IN CONTEXT**

1. **Systematic review**: A comprehensive review of the PubMed literature identified recent advancements in blood‐based biomarkers for Alzheimer's disease (AD), particularly amyloid beta (Aβ) and phosphorylated tau (p‐tau). Although numerous studies have explored these biomarkers individually, few have directly compared the relationships between homocysteine (Hcy), p‐tau, Aβ, and cortical thickness. Relevant studies are cited throughout the article to contextualize and deepen the understanding of this area of research.
2. **Interpretation**: Increased blood levels of Hcy and p‐tau217 were significantly negatively correlated with temporal cortical thickness, especially in Aβ‐positive individuals. Moreover, Hcy and p‐tau217 exhibited a synergistic interaction, with higher Hcy levels amplifying the detrimental effects of p‐tau217 on temporal cortical thinning and cognitive impairment. These findings highlight Hcy as a potential modifiable risk factor for AD.
3. **Future directions**: Longitudinal studies are essential to elucidate the interaction between Hcy and p‐tau217 biomarkers over time and their potential for early diagnosis and therapeutic intervention. In addition, categorizing patients based on Aβ status and conducting clinical trials to reduce Hcy levels could solidify the recognition of Hcy as a modifiable risk factor for AD.

### 2.2 Cognitive evaluation

As described previously, participants underwent a comprehensive neuropsychological evaluation using sensitive tests assessing all cognitive domains^30^: the Mini‐Mental State Examination (MMSE), Clinical Dementia Rating (CDR) scale, activities of daily living (ADLs) scale, Hachinski Ischemic Score (HIS), Auditory Verbal Learning Test (AVLT), Trail Making Test (TMT), Symbol‐Digit Modality Test (SDMT), Boston Naming Test (BNT), Rey‒Osterrieth Complex Figure (ROCF) test, Stroop Color and Word Test (SCWT), Digital Span Test (DST), and Clock Drawing Test (CDT).

### 2.3 Blood sample acquisition

Blood samples from 236 participants were collected into 5 mL polypropylene tubes and promptly transported to the laboratory for processing within 4 h of collection. Following centrifugation at 2000 × *g* for 10 min at 4°C, aliquots of 0.5 mL plasma were transferred into separate polypropylene tubes and stored at −80°C until needed for analysis. The time frame for blood collection was within 3 months of the PET scan.

### 2.4 Serum Hcy concentration measurements

Previous studies have detailed the Hcy measurement procedure.^31^ Serum Hcy concentrations were quantified using an enzyme cycling assay conducted on automated analyzers (AU5800, Beckman Coulter, Brea, CA). All samples were analyzed by a research assistant who was blinded to the participants' group status.

### 2.5 Plasma tau and Aβ biomarkers concentration measurements

The plasma samples were thawed at room temperature on the day of analysis. To avoid the impact of multiple freeze‐thaw cycles, only aliquots that had not been thawed previously were used in this study. The plasma levels of Aβ1‐42, Aβ1‐40, p‐tau181, and p‐tau217 were measured directly from the storage tubes containing 0.5 mL of plasma. This was done using the Lumipulse G β‐Amyloid 1‐40 Plasma, Lumipulse G β‐Amyloid 1‐42 Plasma, Lumipulse G p‐tau 181 Plasma, and Lumipulse G p‐tau 217 Plasma assays on the Lumipulse G 1200 automated platform (Fujirebio). The manufacturer's instructions were followed when carrying out all testing procedures. This included vortexing and centrifugation after thawing to prevent fibrin clots from influencing the results.

### 2.6 *APOE ε4* status

The Lumipulse G ApoE ε4 and Pan‐ApoE assays (Fujirebio) were used sequentially to analyze ApoE ε4 and Pan‐ApoE, respectively. The ApoE proteotype status, which indicates the presence of ApoE ε4 alone (homozygous) or in combination with ApoE ε2 or ApoE ε3 (heterozygous) in human plasma, was determined by calculating the ApoE ε4/Pan‐ApoE ratio. The samples were classified as follows: ‘null’ (indicating the absence of ApoE ε4) if the ratio was less than 5%, ‘heterozygous’ (indicating the presence of ApoE ε4 with either ApoE ε2 or ApoE ε3) if the ratio was equal to or greater than 5% but less than 75%, and ‘homozygous’ (indicating the presence of ApoE  ε4/ ε4 without either ApoE  ε2 or ApoE  ε3) if the ratio was equal to or greater than 75%.^32^

### 2.7 Amyloid‐PET imaging acquisition, visual assessment, and quantitative analysis

A total of 236 participants underwent amyloid PET imaging with 18F‐florbetapir. As described previously,^26^ the PET data were captured using a SIGNA PET magnetic resonance (MR) device or a Siemens PET computed tomography (CT) device 50 min after an injection of 10 ± 1 mCi of 18F‐florbetapir.

PET MR images were acquired over 15 min, using the ordered subset expectation maximization reconstruction algorithm along with time‐of‐flight and point spread function corrections. This procedure involved 28 subsets and six iterations. The PET images were adjusted for attenuation through zero‐echo time sequences. The matrix size was 256 × 256, with a display field of view measuring 46.2 × 30 cm, a slice thickness of 2.78 mm, and a pixel size of 2.8 × 2.8 mm.

PET CT scans were acquired over 15 to 20 min using a three‐dimensional (3D) iterative algorithm (four iterations and 21 subsets) with a matrix size of 336 × 336, a zoom factor of 2.0 mm, a slice thickness of 2.0 mm, and an all‐pass filter. Time‐of‐flight and point spread function algorithms were employed with five iterations and 16 subsets for PET image reconstruction. The matrix size was 336 × 336, with a zoom factor of 2.0, a slice thickness of 2.0 mm, and a Gaussian filter with full width at half‐maximum (FWHM) of 5.0.

Blinded to the participants’ clinical diagnoses and biomarker levels, expert readers visually rated all the PET scans. Following U.S. Food and Drug Administration (FDA) recommendations, scans were classified as “positive” when one or more areas showed increased cortical gray matter signaling, leading to reduced or absent contrast between gray and white matter. Scans were classified as “negative” when the contrast between gray and white matter was clear.

Subsequently, we quantified amyloid depositions within the PET scans. Each participant's PET scan was spatially normalized to a Montreal Neurological Institute (MNI) 152 18F‐florbetapir template using linear and nonlinear transformations. The mean 18F‐florbetapir uptake was measured across the frontal, lateral parietal, and anterior/posterior cingulate cortices. Finally, the 18F‐florbetapir standardized uptake value ratio (SUVR) map was extracted using the whole cerebellum as a reference.

### 2.8 Preprocessing of structural MRI data using FreeSurfer

Structural MRI data were preprocessed using FreeSurfer version 6.0. The raw MRI data were reviewed for quality control, ensuring proper orientation and alignment. The data were then processed through FreeSurfer's preprocessing pipeline, which includes several key steps: skull stripping, bias field correction, tissue classification and segmentation, surface reconstruction, and parcellation. Finally, we extracted parcel‐based cortical thickness, which provides measures of cortical thickness for each of the 62 Desikan–Killiany atlas^33^ regions in each participant's native brain space.

### 2.9 Regions of interest (ROI) definition

Because temporal cortical thickness indicates structural neurodegeneration associated with AD,^34^, ^35^ we jointly determined the inferior, middle, and superior temporal regions as the regions Supplementary of interest (ROI).

### 2.10 Statistical analysis

**Data preprocessing**: Missing values (refer to Supplementary Tables S1 and S2 for the missing rates) were imputed via multiple imputation by chained equations utilizing the “mice” package based on R (version 4.3.2), which is widely adopted in epidemiological and clinical research for handling incomplete datasets. The Tukey method was used to identify values exceeding 1.5 times the interquartile range (IQR) and minimize the impact of outliers on the correlation and regression analyses of blood biomarkers. This process was executed with the “outliers” package (version 3) based on R, which replaces identified outliers with their respective quartile values (Q1 or Q3).

**Data analysis**: Independent‐sample *t*‐tests were performed to assess differences in continuous variables between the amyloid‐positive and amyloid‐negative groups. Chi‐square tests were used to evaluate differences in categorical variables between the groups. The TMT Part B (TMT‐B) scores were reverse‐scored, with lower values indicating poorer executive function, to ensure the consistency of the other scales. Exploratory partial correlation analyses were performed to examine the relationships between Hcy levels and various biomarkers, including plasma biomarkers, cortical thickness, and PET measurements. They were adjusted for sex, age, years of education, cognitive status, and *APOE ε4* status. Confirmatory linear regression models were constructed to evaluate the effects of blood Hcy levels, p‐tau217 levels, and SUVRs on temporal cortical thickness and cognitive function: Temporalcorticalthicknessorcognitivefunction=Hcy+Hcy∗p−tau217+Hcy∗SUVRs. To control type I error due to multiple comparisons, false discovery rate (FDR) correction was applied to all regression analyses. All the statistical analyses were conducted using R version 4.3.2 (R Foundation for Statistical Computing, Vienna, Austria). Statistical significance was defined at *α* = 0.05, and all tests were two‐tailed.

## 3 RESULTS

### 3.1 Characterization of the study population

Table 1 presents all participants' clinical, demographic, and blood biomarker data stratified according to amyloid status. The SCABI cohort consisted of 236 participants with an average age of 68.6 years (SD: 7.17); 29.7% were male, and 78.3% were cognitively impaired, as determined by either a diagnosis of MCI or dementia. Among the 40 participants diagnosed with dementia, 35 were classified as having probable AD, 1 as mixed dementia, 2 as frontotemporal dementia (FTD), and 2 as dementia of unknown etiology.

**TABLE 1:** Clinical and demographic characteristics of all participants based on amyloid status.

	[ALL]	Negative	Positive	
	*N = 236*	*N = 136*	*N = 100*	*p*
Cognitive status				< 0.001^***^
Cognitively unimpaired	51 (21.6%)	42 (30.9%)	9 (9.00%)	
MCI	145 (61.4%)	87 (64.0%)	58 (58.0%)	
Dementia	40 (16.9%)	7 (5.15%)	33 (33.0%)	
Sex (Male %)	70 (29.7%)	39 (28.7%)	31 (31.0%)	0.809
*APOE* ε4 (Carrier %)	62 (26.3%)	24 (17.6%)	38 (38.0%)	0.001^**^
Age, mean (SD)	68.6 (7.17)	68.4 (6.83)	68.8 (7.63)	0.741
Education	10.1 (4.19)	11.1 (3.95)	8.70 (4.14)	< 0.001^***^
ADL	12.9 (2.30)	13.5 (1.25)	11.9 (3.00)	< 0.001^***^
MMSE	20.3 (6.40)	22.9 (4.37)	16.7 (6.98)	< 0.001^***^
HIS	1.79 (0.98)	1.79 (0.95)	1.80 (1.03)	0.920
CDR	0.67 (0.50)	0.50 (0.25)	0.90 (0.64)	< 0.001^***^
Hypertension	79 (33.5%)	43 (31.6%)	36 (36.0%)	0.572
Cardiology	34 (14.4%)	20 (14.7%)	14 (14.0%)	1.000
Diabetes mellitus	34 (14.4%)	23 (16.9%)	11 (11.0%)	0.276
Dyslipidemia	75 (31.8%)	50 (36.8%)	25 (25.0%)	0.076
Blood biomarkers				
Homocysteine	10.8 (2.78)	10.4 (2.55)	11.5 (2.96)	0.003^**^
Aβ1‐40 (pg/mL)	268 (41.9)	272 (42.5)	264 (40.8)	0.165
Aβ1‐42 (pg/mL)	24.5 (4.82)	26.7 (4.30)	21.5 (3.82)	< 0.001^***^
Aβ1‐42/1‐40	0.09 (0.01)	0.10 (0.01)	0.08 (0.01)	< 0.001^***^
p‐Tau181 (pg/mL)	2.96 (1.33)	2.36 (0.94)	3.78 (1.35)	< 0.001^***^
p‐Tau217 (pg/mL)	0.39 (0.42)	0.14 (0.05)	0.73 (0.47)	< 0.001^***^
SUVRs	1.17 (0.20)	1.06 (0.11)	1.33 (0.19)	< 0.001^***^
Memory				
AVLT_N13	13.7 (6.27)	15.6 (5.88)	11.1 (5.85)	< 0.001^***^
AVLT_N4	3.85 (3.19)	4.94 (3.03)	2.36 (2.79)	< 0.001^***^
AVLT_N5	3.29 (3.28)	4.51 (3.09)	1.62 (2.76)	< 0.001^***^
AVLT_N6	2.80 (3.24)	3.79 (3.26)	1.45 (2.70)	< 0.001^***^
Executive function				
TMT‐B	99.4 (54.1)	82.1 (48.6)	123 (52.5)	< 0.001^***^
STROOP	41.3 (7.73)	43.6 (5.87)	38.2 (8.84)	< 0.001^***^
Attention				
SDMT	25.6 (14.1)	30.5 (13.4)	19.0 (12.1)	< 0.001^***^
DST	14.6 (3.67)	15.0 (3.72)	14.0 (3.55)	0.048^*^
Language				
BNT	18.2 (4.87)	19.6 (4.24)	16.3 (5.06)	< 0.001^***^
VFT	11.4 (4.15)	12.6 (4.19)	9.83 (3.54)	< 0.001^***^
Visuospatial skill				
CDT	2.95 (1.02)	3.26 (0.83)	2.54 (1.12)	< 0.001^***^
ROCF	22.2 (8.54)	24.6 (7.08)	18.8 (9.24)	< 0.001^***^

*Note*: Data are presented as n (%) or means（standard deviation）

Abbreviations: Aβ, amyloid beta; ADL, activities of daily living; AVLT N1‐3, Auditory Verbal Learning Test Immediate recall; AVLT N4, Auditory Verbal Learning Test Short‐term delayed recall; AVLT N5, Auditory Verbal Learning Test Long‐term delayed recall; AVLT N6, Auditory Verbal Learning Test Recognition; BNT, Boston Naming Test; CDR, Clinical Dementia Rating; CDT, Clock Drawing Test; DST, Digit Span Test; HIS, Hachinski Ischemic Score; MCI, mild cognitive impairment; MMSE, Mini‐Mental State Examination; p‐tau, phosphorylated tau; ROCF, Rey–Osterrieth Complex Figure; SCWT, Stroop Color and Word Test; SDMT, Symbol‐Digit Modality Test; SUVRs, standardized uptake value ratios; TMT, Trail Making Test; t‐tau, total tau.

*p < 0.05.

**p < 0.01.

***p < 0.001.

There was no statistically significant difference in age or sex across groups (*p* > 0.05). Compared with the Aβ‐negative group, the Aβ‐positive group had less education, a greater proportion of *APOE* ε4 carriers (*p* = 0.001), and exhibited worse ADL function and cognitive performance (*p* < 0.05). No statistically significant differences were found in HIS (1.79 vs 1.80, *p* = 0.920) and in the incidence of hypertension, cardiology, diabetes mellitus, or dyslipidemia (*p* > 0.05).

Hcy levels were significantly higher in the Aβ‐positive group (*p* = 0.003), suggesting a role for Hcy in Aβ‐related pathophysiology. The Aβ1‐42 and Aβ1‐42/1‐40 levels were significantly lower in the Aβ‐positive group than in the Aβ‐negative group (*p* < 0.001). Similarly, the p‐tau217 and p‐tau181 levels were significantly increased in the Aβ‐positive group (*p* < 0.001). In addition, the SUVRs, a marker of brain Aβ deposition, were increased in the Aβ‐positive group (*p* < 0.001).

### 3.2 Increased blood Hcy levels are weakly associated with temporal cortical thinning

**FIGURE 1:**
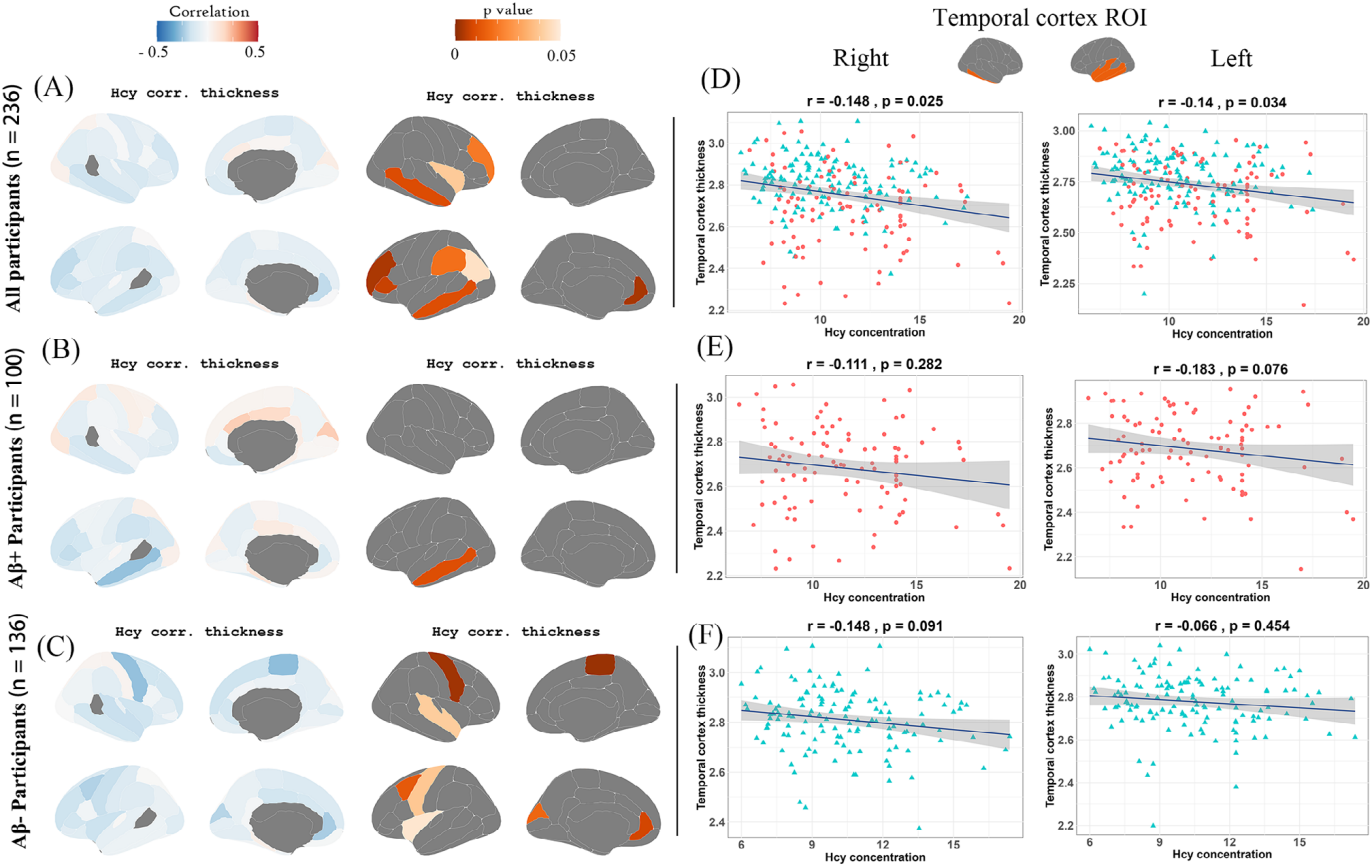
Associations between blood Hcy levels and temporal cortical thickness across Aβ groups (controlling for sex, age, years of education, cognitive status, and *APOE ε4* status). (A–C) The correlations of whole cortical thickness with Hcy. (D–F) The correlations of temporal cortical thickness with Hcy. Aβ, amyloid beta; *APOE*, apolipoprotein E; Hcy, homocysteine.

Blood Hcy levels were negatively correlated with the cortical thickness in 9 of 62 gray matter (GM) regions in the overall cohort (Figure 1A **and** Table S3), 1 of 62 GM regions in the positive group (Figure 1B **and** Table S4), and 8 of 62 GM regions in the negative group (Figure 1C **and** Table S5) (*p* < 0.05). Scatter plots visually highlighted the significant negative correlations in the right temporal cortex (*r* = −0.148, *p* = 0.025) and left temporal cortex (*r* = −0.140, *p* = 0.034) (Figure 1D). In the Aβ‐positive group, negative trends were observed but did not reach statistical significance (right: *r* = −0.111, *p* = 0.282; left: *r* = −0.183, *p* = 0.076) (Figure 1E). Similarly, in the Aβ‐negative group, weak negative correlations were noted but were not statistically significant (right: *r* = −0.148, *p* = 0.091; left: *r* = −0.066, *p* = 0.454) (Figure 1F). These findings suggest that increased Hcy levels are associated with a reduced temporal cortical thickness in the overall cohort, with subgroup analyses showing attenuated correlations.

### 3.3 Increased p‐tau217 levels are strongly associated with temporal cortex thinning

**FIGURE 2:**
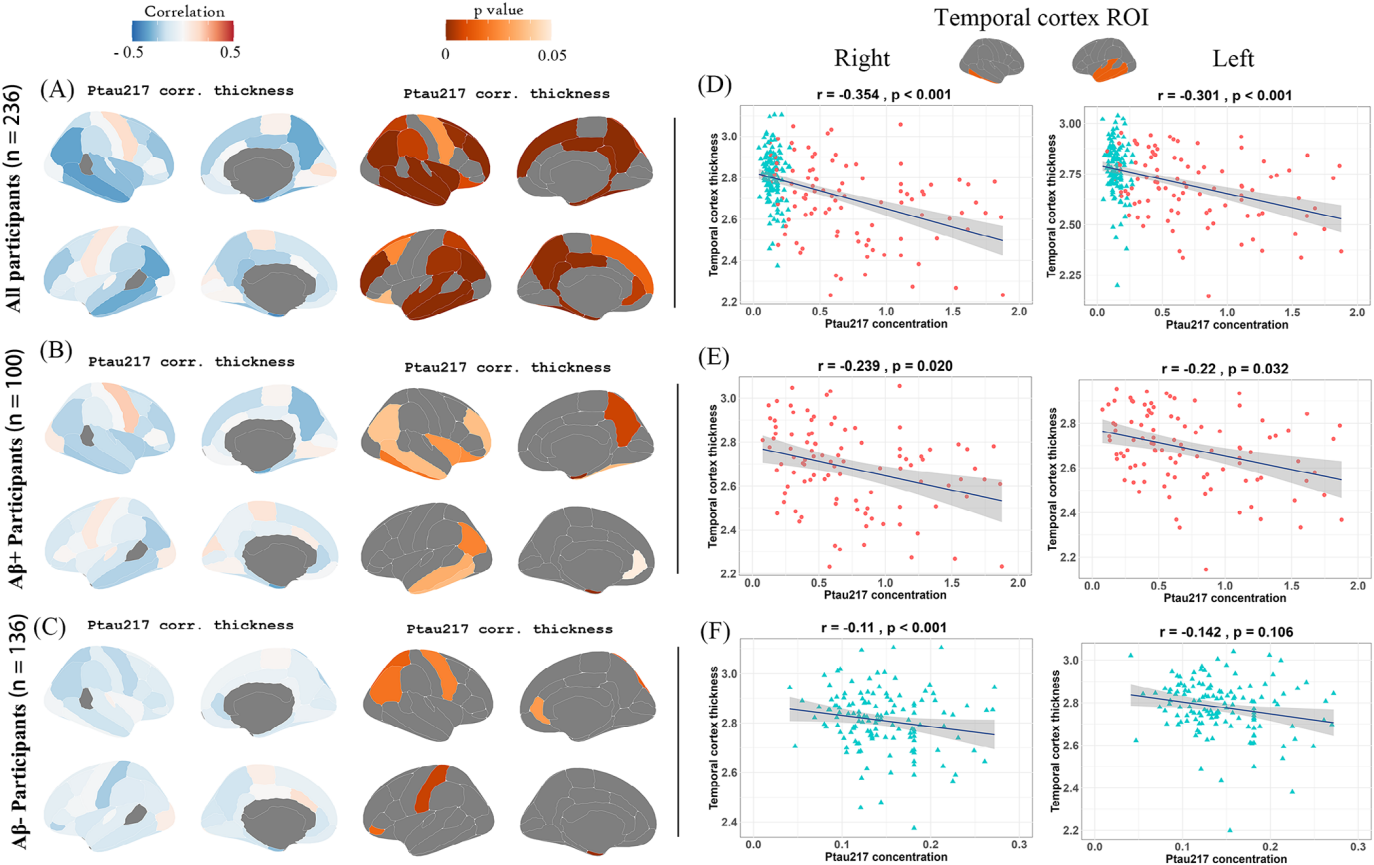
Associations between increased p‐tau217 levels and temporal cortical thickness across Aβ groups (controlling for sex, age, years of education, cognitive status, and *APOE ε4* status). (A–C) The correlations of whole cortical thickness with p‐tau217. (D–F) The correlations of temporal cortical thickness with p‐tau217. Aβ, amyloid beta; *APOE*, apolipoprotein E; Hcy, homocysteine; p‐tau217, phosphorylated tau‐217.

Blood p‐tau217 levels were negatively correlated with cortical thickness in 34 of 62 GM regions (Figure 2A **and** Table S6). In the positive group, a negative correlation was observed in 14 of 62 GM regions (Figure 2B and Table S7). In contrast, in the negative group, the correlation was negative in 7 of 62 GM regions (Figure 2C and Table S8) (*p* < 0.05). Scatter plots revealed robust correlations in the right (*r* = −0.354, *p* < 0.001) and left (*r* = −0.301, *p* < 0.001) temporal cortex thickness (Figure 2D). In Aβ‐positive participants, significant moderate negative correlations were observed in the right (*r* = −0.239, *p* = 0.020) and left (*r* = −0.220, *p* = 0.032) temporal cortical thickness (Figure 2E). In Aβ‐negative participants, weaker negative correlations were observed with the thickness of the right temporal cortex (*r* = −0.110, *p* < 0.001). Still, nonsignificant correlations were detected with the thickness of the left temporal cortex (*r* = −0.142, *p* = 0.106) (Figure 2F).

### 3.4 Blood Hcy levels may contribute to AD pathology depending on the Aβ status

**FIGURE 3:**
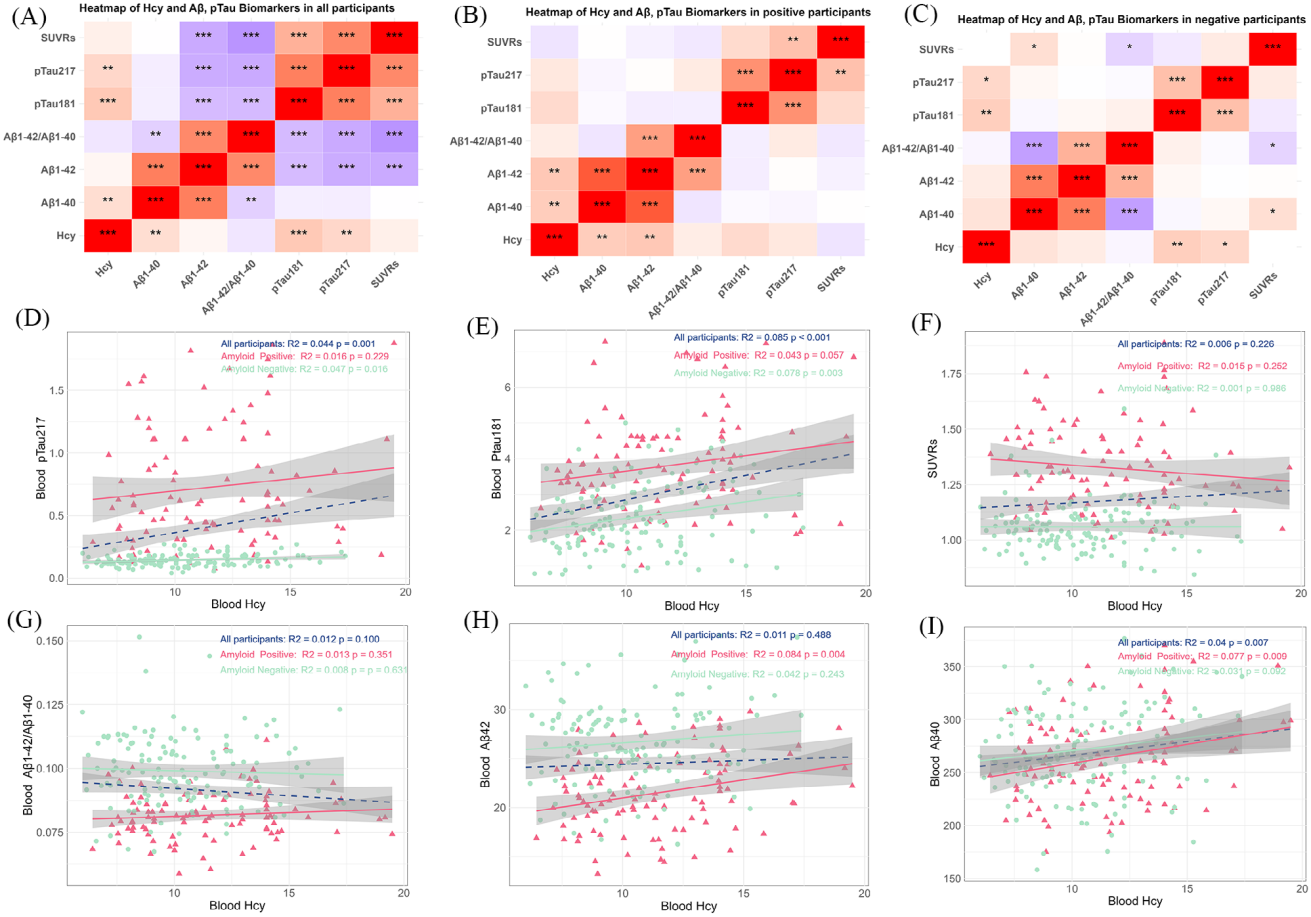
Correlations between blood Hcy levels and AD biomarkers across Aβ groups (FDR corrected, control for sex, age, years of education, cognitive status, and *APOE ε4* status). (A–C) Heatmaps of blood Hcy levels were associated with AD biomarkers across groups. (D–I), Scatterplots and regression between blood Hcy levels and AD biomarkers. Aβ, amyloid beta; AD, Alzheimer's disease; *APOE*, apolipoprotein E; FDR, false discovery rate; Hcy homocysteine; p‐tau, phosphorylated tau; SUVRs, standardized uptake value ratios. *p < 0.05 **p < 0.01, ***p < 0.001.

Heatmaps revealed that, among all participants, blood Hcy levels were associated with p‐tau217 levels, p‐tau181 levels, Aβ1‐40 levels, and SUVRs (Figure 3A) (all *p*_FDRs_ < 0.05). In the Aβ‐positive group, Hcy levels were associated with Aβ1‐40 and Aβ1‐42 levels but not with the Aβ1‐42/Aβ1‐40 ratio or SUVRs (Figure 3B) (all *p*_FDRs_ < 0.05). In the Aβ‐negative group, Hcy levels were associated with p‐tau217 and p‐tau181 levels (Figure 3C) (all *p*_FDRs_ < 0.05). Scatter plots confirmed these findings, showing significant positive correlations between blood Hcy and p‐tau217 (*R* ^2^ = 0.044, *p* = 0.001) and p‐tau181 (*R* ^2^ = 0.085, *p* < 0.001) levels in the overall cohort, with similar correlations for the levels of p‐tau biomarkers in Aβ‐negative participants (p‐tau217: *R* ^2^ = 0.047, *p* = 0.016 and pTau181 *R* ^2^ = 0.078, *p* = 0.003) (Figure 3D,E). In the Aβ‐positive group, blood Hcy levels were significantly associated with Aβ1‐40 and Aβ1‐42 levels (*R* ^2^ = 0.084, *p* < 0.05). In contrast, the associations between blood Hcy levels and the Aβ1‐42/42/1/40 ratio or SUVRs were weak and insignificant across the groups (Figure 3F–II). These findings suggest that blood Hcy levels may contribute to tau pathology, with variable associations with Aβ biomarkers, depending on the Aβ status.

### 3.5 Higher Hcy levels may amplify the adverse effects of p‐tau217 on the temporal cortical thickness

**FIGURE 4:**
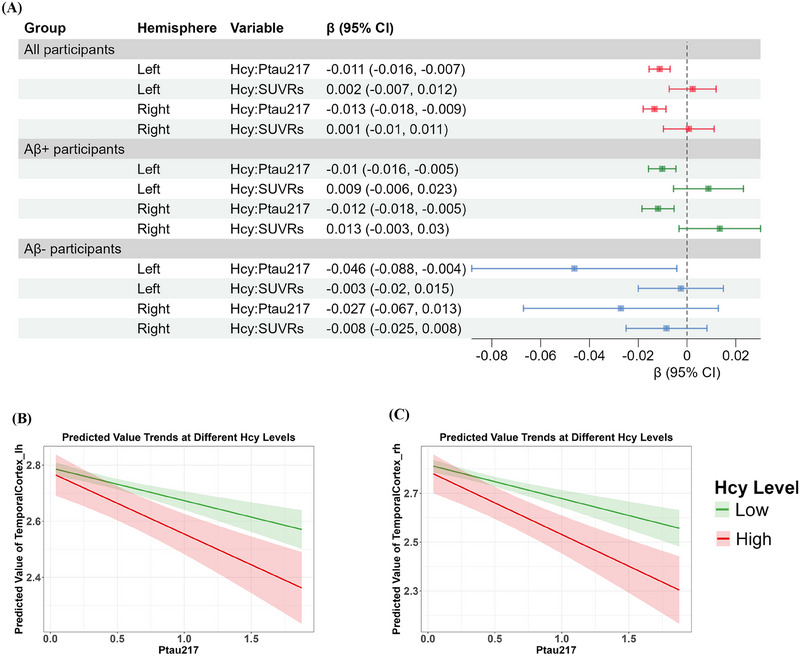
The interaction effect of Hcy and p‐tau217 on temporal cortex thickness thinning across groups (adjusted for sex, *APOE ε4* status, cognitive status, age, and years of education). (A) The interaction effect of Hcy and p‐tau217 on temporal cortex thickness thinning across groups. (B) The trend of the predicted values of the left temporal cortex as a function of p‐tau217 at different Hcy levels. (C) The trend of the predicted values of the right temporal cortex as a function of p‐tau217 at different Hcy levels. Aβ, amyloid beta; *APOE*, apolipoprotein E; CI, confidence interval, Hcy, homocysteine; lh, left hemisphere; p‐tau, phosphorylated tau; rh, right hemisphere; SUVRs, standardized uptake value ratios.

Linear regression models were fitted to explore the effects of the interactions of blood Hcy, p‐tau217, and SUVRs on the temporal cortical thickness after adjusting for covariates, including sex, *APOE ε4* status, cognitive status, age, and years of education (Figure 4A and Table S9). For all participants, the interaction effect between Hcy and p‐tau217 was significantly associated with both the left and right temporal cortex thickness (left: *β* = −0.012, 95% confidence interval [CI] = −0.016 to −0.007; right: *β* = −0.013, 95% CI = −0.018 to −0.009). In contrast, no significant effects were observed for the Hcy and SUVR interaction. In the Aβ‐positive group, similar significant interactions between Hcy and p‐tau217 levels were observed (left: *β* = −0.01, 95% CI = −0.016 to −0.005; right: *β* = −0.012, 95% CI = −0.018 to −0.005). For the Aβ‐negative group, the interaction effect between Hcy and p‐tau217 levels was insignificant in the temporal cortical thickness (*p*_FDRs_ > 0.05).

Hcy levels were dichotomized into high and low groups based on the median, and the predicted values of temporal cortical thickness were plotted further to illustrate the interaction (Figure 4B,C). Among all participants, higher Hcy levels were associated with a more pronounced inverse relationship between p‐tau217 levels and temporal cortical thickness, suggesting that increased Hcy levels amplify the negative impact of p‐tau217 pathology on the temporal cortex. In contrast, at lower Hcy levels, the effect of p‐tau217 on cortical thickness was less pronounced, as indicated by a flatter curve, implying a reduced influence of p‐tau217 pathology. These findings underscore that increased Hcy levels may amplify the adverse effects of p‐tau217 on the temporal cortical thickness.

### 3.6 Increased Hcy levels may amplify the adverse effects of p‐tau217 on cognitive impairment

**FIGURE 5:**
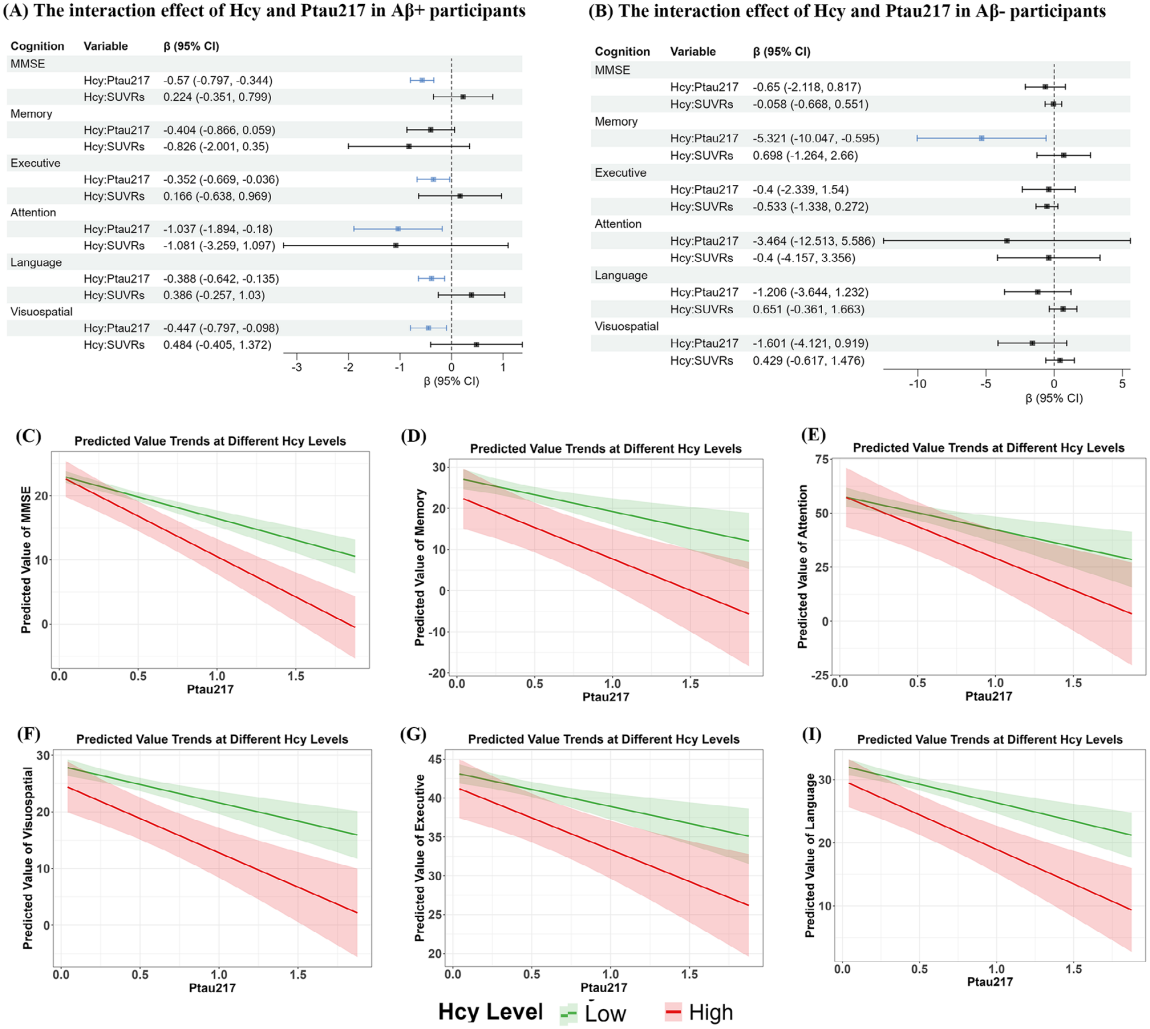
The interaction effect of Hcy and p‐tau217 on specific cognitive domains across groups (adjusted for sex, *APOE ε4* status, cognitive status, age, and years of education). (A) The interaction effect of Hcy and p‐tau217 on specific cognitive domains thinning Aβ‐positive participants. (B) The interaction effect of Hcy and p‐tau217 on specific cognitive domains thinning Aβ‐negative participants. (C–I) The trend of the predicted values of the specific cognitive domains as a function of p‐tau217 at different Hcy levels. Aβ, amyloid beta; *APOE*, apolipoprotein E; CI, confidence interval; Hcy, homocysteine; p‐tau, phosphorylated tau; SUVRs, standardized uptake value ratios.

Figure 5 shows a significant interaction effect of Hcy and p‐tau217 levels on specific cognitive domains in Aβ‐positive and Aβ‐negative participants. The interaction of Hcy and p‐tau217 levels was significantly negatively associated with global cognition (MMSE) (*β* = −0.57, 95% CI = −0.797 to −0.344), executive function (*β* = −0.352, 95% CI = −0.669 to −0.036), attention (*β* = −1.037, 95% CI = −1.894 to −0.18), language (*β* = −0.388, 95% CI = −0.642to −0.135), and visuospatial function (*β* = −0.447, 95% CI = −0.797 to −0.098). Similar results were observed among all participants (Figure S2). However, the interaction of Hcy and p‐tau217 levels was only significantly negatively associated with memory (*β* = −5.321, 95% CI = −10.047, −0.595) in Aβ‐negative participants (Figure 5B).

Hcy levels were dichotomized into high and low groups based on the media, and the predicted values of specific cognitive domains were plotted to further illustrate the interaction (Figure 5C–H). In all participants, at higher Hcy levels, the decline in cognitive function was more pronounced with increasing p‐tau217 levels, indicating that increased Hcy levels exacerbate the negative impact of tau pathology on cognitive impairment. In contrast, at lower Hcy levels, the effect of p‐tau217 on cognitive function was less pronounced, suggesting a diminished influence of tau pathology. These findings highlight that increased Hcy levels may amplify the adverse effects of p‐tau217 on cognitive impairment.

### 3.7 Sensitivity analyses

Sensitivity analyses were performed to confirm the robustness of the results as follows: (1) Adjusting the significance threshold to a more stringent *p*‐value of < 0.01 confirmed the robustness of the effect of the interaction between Hcy and p‐tau217 levels on temporal cortical thinning (Table S9). (2) A reanalysis excluding missing data produced results consistent with those obtained via imputation (Table S10). (3) Separate analyses of males and females demonstrated no distinction in the results (Table S11 and S12).

## 4 DISCUSSION

This study provides new insights into the associations and interactive effects of blood Hcy and p‐tau217 levels with temporal cortical thickness, cognitive function, and AD pathology. The key results can be summarized as follows: (1) Hcy levels were negatively correlated with the temporal cortical thickness in all participants. (2) p‐Tau217 levels were negatively correlated with bilateral temporal cortical thickness in all participants and the Aβ‐positive group. In contrast, in the Aβ‐negative group, the association was observed only with right temporal cortical thickness. (3) Notably, Hcy and p‐tau217 levels exhibited an significant interaction effect, where higher Hcy levels were associated with a greater adverse impact of p‐tau217 on temporal cortical thinning and cognitive impairment. (4) Hcy levels were strongly associated with tau pathology (p‐tau217 and p‐tau181) in Aβ‐negative individuals. These findings underscore the interactive contributions of Hcy and p‐tau217 to AD progression, highlighting that Hcy may be a modifiable risk factor for AD.

### 4.1 Interaction effects of Hcy and p‐tau217 on temporal cortical thinning and cognitive impairment

This study revealed a significant interaction between blood Hcy and p‐tau217 levels, with higher Hcy levels potentially amplifying the adverse effects of p‐tau217 on temporal cortical thinning and cognitive deficits, particularly in Aβ‐positive individuals. Consistent with prior research,^11^ our findings further corroborate the association between Hcy and neurodegenerative biomarkers. Mechanistically, Hcy may influence Aβ and tau metabolism through various pathways, including involvement in methylation reactions, neuroinflammation, and oxidative stress.^36^, ^37^, ^38^, ^39^, ^40^ Notably, in the early stages of AD, increased Hcy exacerbates Aβ‐induced cerebral endothelial cell (cEC) apoptosis, blood–brain barrier dysfunction, and angiogenic impairment.^41^ Conversely, the accumulation of Aβ may also impact Hcy metabolism by altering the cellular redox state, DNA methylation patterns, and gene transcription.^42^ This study underscores the intricate and potentially synergistic interplay between Hcy and core AD pathologies, warranting further investigation into their combined contributions to disease progression.

However, the *β*‐values for cortical thinning were very low, suggesting that the effect of the Hcy/p‐tau217 interaction on cortical thickness is subtle. This result may indicate that although Hcy and p‐tau217 interact to influence neurodegeneration, their collective impact on cortical thickness may be subtle and influenced by other factors not accounted for. These findings underscore the importance of understanding the relationship between Hcy and tau pathology as interconnected processes in AD, highlighting the complexity of its pathology. Despite the low *β*‐values, the interaction between Hcy and p‐tau217 remains an essential avenue for further exploration, as it may point to more nuanced mechanisms of neurodegeneration.

### 4.2 p‐tau217 as a key driver of temporal cortical thinning

This study confirms a robust association between plasma p‐tau217 levels and temporal cortical thinning. In both the overall cohort and the Aβ‐positive subgroup, higher p‐tau217 levels were significantly associated with cortical thinning in both hemispheres, reinforcing its role as a key marker of tau pathology and neuronal damage in individuals with AD.^43^, ^44^ Compared with Hcy levels, the stronger correlation between p‐tau217 levels and cortical thinning underscores the central role of tau pathology in AD‐related neurodegeneration. This finding is consistent with previous studies showing that phosphorylated tau species, particularly p‐tau217, are closely linked to neuronal injury and predict AD progression.^45^, ^46^ In contrast, weaker but significant correlations were observed in the right temporal cortex of the Aβ‐negative group. These findings suggest that tau pathology in this population may involve non–amyloid‐dependent mechanisms, such as oxidative stress or inflammation.^47^ In summary, these findings emphasize the importance of the Aβ status in modulating the relationship between tau pathology and cortical thinning, and highlight p‐tau217 as a promising biomarker for assessing tau‐driven neurodegeneration in diverse pathological contexts.

### 4.3 Amyloid‐specific attenuation of the impact of Hcy on temporal cortical thinning and AD biomarkers

The present results suggest that increased blood Hcy levels significantly correlate with reduced temporal cortical thickness in all participants; however, this association was not statistically significant in the Aβ‐positive or Aβ‐negative subgroups. In Aβ‐positive individuals, Aβ deposition and its neurotoxic effects may dominate or modulate the impact of Hcy.^48^ Conversely, in Aβ‐negative individuals, alternative protective mechanisms or compensatory processes, such as reduced neuroinflammation or enhanced vascular integrity,^36^, ^49^ may mitigate the effects of Hcy. The observed Aβ‐specific differences indicate that the impact of Hcy on cortical thinning is contingent upon coexisting pathologies, particularly tau aggregation, rather than functioning as an independent factor in neurodegeneration.

Similarly, the findings indicate that in the context of Aβ pathology, blood Hcy levels are more strongly associated with tau biomarkers (p‐tau217 and p‐tau181) than with Aβ markers, such as SUVRs and the Aβ42/40 ratio. This observation aligns with prior research suggesting that Hcy may primarily drive tau‐related pathology rather than Aβ aggregation.^9^, ^12^ These Aβ‐specific differences indicate that the influence of Hcy on AD progression may depend on coexisting pathologies, particularly tau aggregation. Further investigations are warranted to elucidate these interactions and their implications for AD progression.

### 4.4 Clinical implications

The findings of this study have several important clinical implications. (1) Hcy may serve as a modifiable risk factor: Interventions aimed at reducing Hcy levels, such as folate or vitamin B12 supplementation, may hold therapeutic potential for mitigating tau‐related cortical thinning. Previous interventional studies have shown that lowering Hcy through vitamin supplementation can effectively reduce Hcy levels^50^ and improve cognitive function.^51^ However, some studies argue that this approach does not consistently prevent cognitive decline.^52^ These discrepancies may be influenced by Aβ status, particularly in Aβ‐positive individuals, where the independent effect of Hcy appears weaker. These findings suggest that combined interventions targeting Aβ and tau pathology may be necessary. (2) p‐Tau217 is a diagnostic and prognostic marker. Plasma p‐tau217 levels, owing to their strong association with cortical thinning, represent a promising biomarker for early diagnosis and monitoring AD progression. Recent studies have shown that p‐tau217 levels strongly correlate with Aβ and tau pathology and cognitive decline, making it a particular and sensitive biomarker for differentiating AD from other neurodegenerative diseases.^25^, ^53^ (4) The significant interaction between Hcy and p‐tau217 highlights the need for personalized treatment approaches that address tau pathology and vascular/metabolic factors in individuals with AD. Such strategies could improve therapeutic efficacy by accounting for the unique pathological mechanisms contributing to disease progression.

### 4.5 Limitations and future directions

However, the cross‐sectional design of this study limits causal inferences, leaving unresolved questions about whether increased Hcy levels drive neurodegeneration or result from other pathological processes. Although participants were stratified by their Aβ status, potential confounding factors, such as diet and lifestyle, were not considered. Blood samples were collected after an 8‐h fast, which may have influenced Hcy's metabolism. In addition, although plasma p‐tau biomarkers are clinically accessible, they may not fully capture localized p‐tau‐mediated processes in the brain. Finally, the inclusion criterion for folate or vitamin B12 deficiency relied on self‐reports (yes/no) rather than laboratory tests, potentially leading to the misidentification of folate deficiency in some participants. Future studies should incorporate longitudinal data, advanced neuroimaging, cerebrospinal biomarkers, and more precise testing methods to ensure these findings' accuracy, reliability, and validation.

## 5 CONCLUSIONS

The present study showed that increased Hcy and p‐tau217 levels are independent and interact with temporal cortical thinning and cognitive impairment, particularly in Aβ‐positive individuals. These findings emphasize the importance of integrating metabolic and tau‐related pathways for a comprehensive understanding AD. Hcy may serve as a modifiable risk factor, whereas p‐tau217 has emerged as a superior diagnostic biomarker and potential therapeutic target. Together, these insights lay the foundation for developing novel diagnostic and therapeutic strategies for AD.

## CONFLICT OF INTEREST STATEMENT

The authors declare that they have no conflicts of interest. Any author disclosures are available in the Supporting Information.

## CONSENT STATEMENT

All participants or their legal guardians provided signed informed consent to participate in the study. The study adhered to the Declaration of Helsinki and was approved by the Affiliated Brain Hospital Ethics Committees of Guangzhou Medical University.

## Supporting information



Supporting Information

Supporting Information
