# Supplementary material for: Association and interaction of blood homocysteine and p‐tau217 levels with temporal cortical thinning and cognitive impairment in Alzheimer's disease
Source: Alzheimers Dement. 2025 Jul 17;21(7):e70465. doi: 10.1002/alz.70465 (PMC12268315; doi:10.1002/alz.70465)
Supplement: Supplementary file 1 — Supporting Information [file ALZ-21-e70465-s001.docx]

**Supplementary materials**

[Supplementary Figures 3](#_Toc198756604)

[**Figure S1. Flowchart of participant enrolment.** 3](#_Toc198756605)

[**Figure S2. The interaction effect of Hcy and Ptau217 on specific cognitive domains in all participants** (adjusted for sex, APOE status, cognitive status, age, and education). Abbreviations: Hcy, homocysteine; Ptau, phosphorylated tau, SUVRs, standardized uptake value ratios. 4](#_Toc198756606)

[Supplementary Tables 5](#_Toc198756607)

[**Table S1. The numbers (percentages) of participants with missing values in positive participants (n=100).** 5](#_Toc198756608)

[**Table S2. The numbers (percentages) of participants with missing values in negative participants (n=136).** 5](#_Toc198756609)

[**Table S3. Correlation results between homocysteine and brain cortex thickness in all participants.** 6](#_Toc198756610)

[**Table S4. Correlation results between homocysteine and brain cortex thickness in Aβ-positive groups.** 7](#_Toc198756611)

[**Table S5. Correlation results between homocysteine and brain cortex thickness in Aβ-negative groups.** 8](#_Toc198756612)

[**Table S6. Correlation results between Ptau217 and brain cortex thickness in all participants.** 10](#_Toc198756613)

[**Table S7. Correlation results between pTau217 and brain cortex thickness in Aβ-positive groups.** 11](#_Toc198756614)

[**Table S8. Correlation results between pTau217 and brain cortex thickness in Aβ-negative groups.** 12](#_Toc198756615)

[**Table S9. Linear regression results for temporal cortex thickness in all participants, Aβ-positive and Aβ-negative groups.** (adjusted for sex, APOE status, cognitive status, age, and education) 13](#_Toc198756616)

[**Table S10. Linear regression results for temporal cortex thickness in all participants, Aβ-positive and Aβ-negative groups excluding missing data. (**adjusted for sex, APOE status, cognitive status, age, and education) (N = 220) 14](#_Toc198756617)

[**Table S11. Linear regression results for temporal cortex thickness in all participants, Aβ-positive and Aβ-negative groups with males. (**adjusted for APOE status, cognitive status, age, and education) (N = 70) 17](#_Toc198756618)

[**Table S12. Linear regression results for temporal cortex thickness in all participants, Aβ-positive and Aβ-negative groups with females.** (adjusted for APOE status, cognitive status, age, and education) (N = 166) 18](#_Toc198756619)

# **Supplementary Figures**


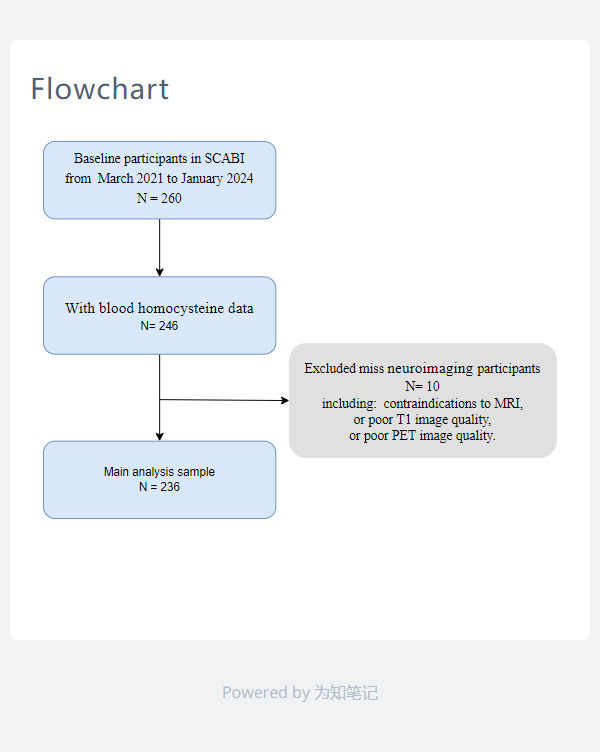


**Figure S1. Flowchart of participant enrolment.**

**
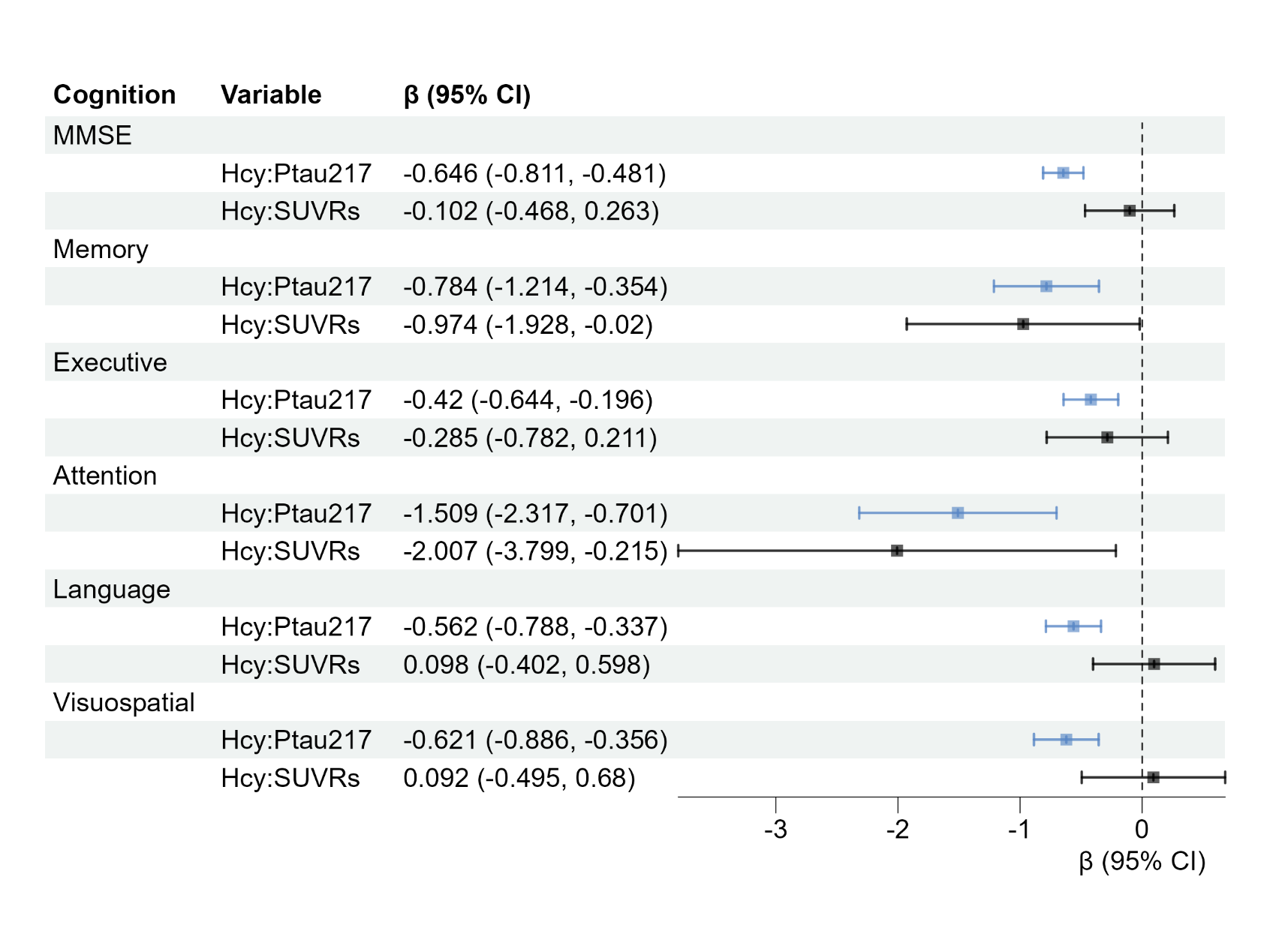
**

**Figure S2. The interaction effect of Hcy and Ptau217 on specific cognitive domains in all participants** (adjusted for sex, APOE status, cognitive status, age, and education). Abbreviations: Hcy, homocysteine; Ptau, phosphorylated tau, SUVRs, standardized uptake value ratios.

# **Supplementary Tables**

## **Table S1. The numbers (percentages) of participants with missing values in positive participants (n=100).**

| **Covariates** | **n** | **%** |
| --- | --- | --- |
| Hcy | 0 | 0 |
| Aβ1-40(pg/ mL) | 1 | 1.0 |
| Aβ1-42(pg/ mL) | 0 | 0 |
| pTau181(pg/ mL) | 0 | 0 |
| pTau217(pg/ mL) | 0 | 0 |
| ApoE4 | 9 | 9.0 |
| SUVRs | 0 | 0 |
| Education | 1 | 1.0 |

Abbreviations: Aβ, amyloid β; pTau, phosphorylated tau; tTau, total tau; SUVRs, Standardized Uptake Value Ratios.

## **Table S2. The numbers (percentages) of participants with missing values in negative participants (n=136).**

| **Covariates** | **n** | **%** |
| --- | --- | --- |
| Hcy | 0 | 0 |
| Aβ1-40(pg/ mL) | 1 | 0 |
| Aβ1-42(pg/ mL) | 0 | 0 |
| pTau181(pg/ mL) | 0 | 9 |
| pTau217(pg/ mL) | 0 | 0 |
| ApoE4 | 7 | 5.15 |
| SUVRs | 0 | 0 |

Abbreviations: Aβ, amyloid β; pTau, phosphorylated tau; tTau, total tau; SUVRs, Standardized Uptake Value Ratios.

## **Table S3. Correlation results between homocysteine and brain cortex thickness in all participants.**

|  | Left hemisphere | |  | Right hemisphere | |
| --- | --- | --- | --- | --- | --- |
| Brain region | Correlation | p_value |  | Correlation | p_value |
| Caudal anterior cingulate | -0.078 | 0.239 |  | 0.067 | 0.314 |
| Caudal middle frontal | -0.122 | 0.065 |  | -0.063 | 0.342 |
| Cuneus | -0.094 | 0.153 |  | 0.062 | 0.348 |
| Entorhinal | -0.051 | 0.441 |  | -0.035 | 0.601 |
| Fusiform | -0.063 | 0.338 |  | -0.063 | 0.337 |
| Inferior parietal | -0.131 | 0.047* |  | -0.097 | 0.143 |
| Inferior temporal | -0.122 | 0.064 |  | -0.114 | 0.084 |
| Isthmus cingulate | -0.065 | 0.324 |  | -0.057 | 0.387 |
| Lateral occipital | -0.026 | 0.698 |  | 0.040 | 0.547 |
| Lateral orbitofrontal | -0.101 | 0.126 |  | -0.108 | 0.102 |
| lingual | -0.044 | 0.508 |  | -0.023 | 0.730 |
| Medial orbitofrontal | -0.006 | 0.934 |  | -0.120 | 0.068 |
| Middle temporal | -0.170 | 0.010** |  | -0.171 | 0.009** |
| Parahippocampal | 0.053 | 0.427 |  | -0.030 | 0.655 |
| Paracentral | -0.037 | 0.571 |  | -0.125 | 0.057 |
| Parsopercularis | -0.073 | 0.271 |  | -0.021 | 0.755 |
| Parsorbitalis | -0.126 | 0.057 |  | -0.083 | 0.210 |
| Parstriangularis | -0.178 | 0.007** |  | -0.098 | 0.136 |
| Pericalcarine | -0.046 | 0.486 |  | -0.022 | 0.744 |
| Postcentral | -0.098 | 0.139 |  | -0.009 | 0.887 |
| Posteriorcingulate | -0.080 | 0.225 |  | 0.018 | 0.791 |
| Precentral | -0.096 | 0.146 |  | -0.107 | 0.106 |
| Precuneus | -0.102 | 0.121 |  | -0.094 | 0.153 |
| Rostral anterior cingulate | -0.193 | 0.003** |  | -0.103 | 0.119 |
| Rostral middle frontal | -0.192 | 0.003** |  | -0.153 | 0.020* |
| Superior frontal | -0.081 | 0.220 |  | -0.090 | 0.171 |
| Superior parietal | 0.005 | 0.940 |  | 0.029 | 0.659 |
| Superior temporal | -0.075 | 0.255 |  | -0.107 | 0.103 |
| Supramarginal | -0.156 | 0.018** |  | -0.081 | 0.222 |
| Transverse temporal | -0.006 | 0.934 |  | -0.018 | 0.787 |
| Insula | -0.107 | 0.105 |  | -0.135 | 0.040** |

Adjusted for sex, APOE status, cognitive status, age, and education

## **Table S4. Correlation results between homocysteine and brain cortex thickness in Aβ-positive groups.**

|  | Left hemisphere | |  | Right hemisphere | |
| --- | --- | --- | --- | --- | --- |
| Brain region | Correlation | p_value |  | Correlation | p_value |
| Caudal anterior cingulate | 0.032 | 0.762 |  | 0.191 | 0.063 |
| Caudal middle frontal | -0.038 | 0.714 |  | 0.037 | 0.721 |
| Cuneus | 0.033 | 0.749 |  | 0.193 | 0.061 |
| Entorhinal | -0.014 | 0.892 |  | 0.004 | 0.971 |
| Fusiform | -0.091 | 0.380 |  | -0.055 | 0.598 |
| Inferior parietal | -0.138 | 0.181 |  | -0.083 | 0.427 |
| Inferior temporal | -0.165 | 0.110 |  | -0.125 | 0.227 |
| Isthmus cingulate | -0.048 | 0.647 |  | 0.063 | 0.545 |
| Lateral occipital | 0.045 | 0.662 |  | 0.096 | 0.357 |
| Lateral orbitofrontal | -0.094 | 0.366 |  | -0.101 | 0.332 |
| lingual | -0.018 | 0.862 |  | 0.012 | 0.907 |
| Medial orbitofrontal | 0.024 | 0.815 |  | -0.124 | 0.230 |
| Middle temporal | -0.264 | 0.010** |  | -0.145 | 0.161 |
| Parahippocampal | 0.111 | 0.285 |  | 0.056 | 0.591 |
| Paracentral | 0.022 | 0.833 |  | 0.022 | 0.830 |
| Parsopercularis | -0.152 | 0.141 |  | -0.017 | 0.868 |
| Parsorbitalis | -0.159 | 0.123 |  | -0.114 | 0.270 |
| Parstriangularis | -0.187 | 0.070 |  | -0.085 | 0.411 |
| Pericalcarine | -0.035 | 0.739 |  | 0.016 | 0.878 |
| Postcentral | -0.091 | 0.378 |  | -0.051 | 0.625 |
| Posterior cingulate | 0.071 | 0.493 |  | 0.154 | 0.137 |
| Precentral | -0.050 | 0.633 |  | 0.050 | 0.628 |
| Precuneus | -0.075 | 0.467 |  | -0.040 | 0.697 |
| Rostral anterior cingulate | -0.132 | 0.201 |  | -0.023 | 0.826 |
| Rostral middle frontal | -0.166 | 0.109 |  | -0.117 | 0.257 |
| Superior frontal | -0.035 | 0.735 |  | 0.027 | 0.799 |
| Superior parietal | 0.047 | 0.651 |  | 0.078 | 0.450 |
| Superior temporal | -0.047 | 0.651 |  | -0.023 | 0.822 |
| Supramarginal | -0.168 | 0.103 |  | -0.029 | 0.782 |
| Transverse temporal | 0.010 | 0.926 |  | 0.001 | 0.994 |
| Insula | -0.056 | 0.590 |  | -0.081 | 0.437 |

Adjusted for sex, APOE status, cognitive status, age, and education

## **Table S5. Correlation results between homocysteine and brain cortex thickness in Aβ-negative groups.**

|  | Left hemisphere | |  | Right hemisphere | |
| --- | --- | --- | --- | --- | --- |
| Brain region | Correlation | p_value |  | Correlation | p_value |
| Caudal anterior cingulate | -0.139 | 0.113 |  | -0.033 | 0.709 |
| Caudal middle frontal | -0.218 | 0.013** |  | -0.160 | 0.067 |
| Cuneus | -0.211 | 0.016** |  | -0.017 | 0.849 |
| Entorhinal | -0.045 | 0.609 |  | 0.001 | 0.987 |
| Fusiform | -0.026 | 0.768 |  | -0.014 | 0.870 |
| Inferior parietal | -0.063 | 0.476 |  | -0.041 | 0.645 |
| Inferior temporal | -0.047 | 0.591 |  | -0.049 | 0.578 |
| Isthmus cingulate | -0.018 | 0.836 |  | -0.099 | 0.259 |
| Lateral occipital | -0.104 | 0.235 |  | 0.001 | 0.988 |
| Lateral orbitofrontal | -0.096 | 0.275 |  | -0.100 | 0.256 |
| lingual | -0.089 | 0.314 |  | -0.053 | 0.545 |
| Medial orbitofrontal | -0.013 | 0.882 |  | -0.085 | 0.334 |
| Middle temporal | -0.050 | 0.572 |  | -0.160 | 0.067 |
| Parahippocampal | 0.050 | 0.569 |  | -0.065 | 0.460 |
| Paracentral | -0.103 | 0.242 |  | -0.275 | 0.001** |
| Parsopercularis | -0.030 | 0.735 |  | -0.032 | 0.720 |
| Parsorbitalis | -0.099 | 0.260 |  | -0.033 | 0.705 |
| Parstriangularis | -0.160 | 0.068 |  | -0.089 | 0.310 |
| Pericalcarine | -0.083 | 0.347 |  | -0.115 | 0.191 |
| Postcentral | -0.116 | 0.189 |  | 0.028 | 0.748 |
| Posterior cingulate | -0.159 | 0.070 |  | -0.054 | 0.539 |
| Precentral | -0.180 | 0.039* |  | -0.264 | 0.002** |
| Precuneus | -0.067 | 0.447 |  | -0.092 | 0.295 |
| Rostral anterior cingulate | -0.232 | 0.008 |  | -0.159 | 0.069 |
| Rostral middle frontal | -0.170 | 0.052 |  | -0.157 | 0.072 |
| Superior frontal | -0.120 | 0.172 |  | -0.170 | 0.053 |
| Superior parietal | 0.002 | 0.984 |  | 0.024 | 0.787 |
| Superior temporal | -0.077 | 0.384 |  | -0.179 | 0.041* |
| Supramarginal | -0.108 | 0.221 |  | -0.095 | 0.281 |
| Transverse temporal | 0.007 | 0.936 |  | -0.064 | 0.467 |
| Insula | -0.173 | 0.048* |  | -0.165 | 0.059 |

Adjusted for sex, APOE status, cognitive status, age, and education

## **Table S6. Correlation results between Ptau217 and brain cortex thickness in all participants.**

|  | Left hemisphere | |  | Right hemisphere | |
| --- | --- | --- | --- | --- | --- |
| Brain region | Correlation | p_value |  | Correlation | p_value |
| Caudal anterior cingulate | -0.004 | 0.955 |  | -0.001 | 0.989 |
| Caudal middle frontal | -0.149 | 0.024* |  | -0.208 | 0.001** |
| Cuneus | 0.052 | 0.433 |  | -0.073 | 0.267 |
| Entorhinal | -0.400 | <0.001*** |  | -0.415 | <0.001*** |
| Fusiform | -0.258 | <0.001*** |  | -0.301 | <0.001*** |
| Inferior parietal | -0.344 | <0.001*** |  | -0.345 | <0.001*** |
| Inferior temporal | -0.254 | <0.001*** |  | -0.304 | <0.001*** |
| Isthmus cingulate | -0.259 | <0.001*** |  | -0.218 | 0.001** |
| Lateral occipital | 0.001 | 0.991 |  | -0.032 | 0.628 |
| Lateral orbitofrontal | -0.137 | 0.037* |  | -0.172 | 0.009** |
| lingual | 0.019 | 0.773 |  | 0.015 | 0.819 |
| Medial orbitofrontal | -0.078 | 0.238 |  | -0.107 | 0.104 |
| Middle temporal | -0.327 | <0.001*** |  | -0.357 | <0.001*** |
| Parahippocampal | -0.208 | 0.001** |  | -0.224 | 0.001** |
| Paracentral | 0.101 | 0.127 |  | 0.042 | 0.527 |
| Parsopercularis | -0.025 | 0.710 |  | -0.114 | 0.084 |
| Parsorbitalis | -0.099 | 0.135 |  | -0.126 | 0.055 |
| Parstriangularis | -0.093 | 0.158 |  | -0.025 | 0.703 |
| Pericalcarine | -0.006 | 0.923 |  | 0.127 | 0.053 |
| Postcentral | -0.030 | 0.653 |  | -0.016 | 0.805 |
| Posterior cingulate | -0.218 | 0.001*** |  | -0.251 | <0.001*** |
| Precentral | 0.086 | 0.191 |  | 0.146 | 0.027*** |
| Precuneus | -0.222 | 0.001*** |  | -0.326 | <0.001*** |
| Rostral anterior cingulate | -0.183 | 0.005** |  | -0.060 | 0.366 |
| Rostral middle frontal | -0.244 | <0.001*** |  | -0.261 | <0.001*** |
| Superior frontal | -0.160 | 0.015** |  | -0.225 | 0.001** |
| Superior parietal | -0.182 | 0.006** |  | -0.181 | 0.006*** |
| Superior temporal | -0.215 | 0.001** |  | -0.279 | <0.001*** |
| Supramarginal | -0.200 | 0.002** |  | -0.181 | 0.006** |
| Transverse temporal | 0.013 | 0.843 |  | -0.083 | 0.211 |
| Insula | -0.092 | 0.165 |  | -0.265 | <0.001*** |

Adjusted for sex, APOE status, cognitive status, age, and education

## **Table S7. Correlation results between pTau217 and brain cortex thickness in Aβ-positive groups.**

|  | Left hemisphere | |  | Right hemisphere | |
| --- | --- | --- | --- | --- | --- |
| Brain region | Correlation | p_value |  | Correlation | p_value |
| Caudal anterior cingulate | 0.062 | 0.549 |  | 0.006 | 0.956 |
| Caudal middle frontal | -0.095 | 0.360 |  | -0.157 | 0.128 |
| Cuneus | 0.116 | 0.262 |  | -0.092 | 0.377 |
| Entorhinal | -0.336 | 0.001** |  | -0.332 | 0.001** |
| Fusiform | -0.181 | 0.079 |  | -0.215 | 0.036* |
| Inferior parietal | -0.234 | 0.023* |  | -0.212 | 0.039* |
| Inferior temporal | -0.215 | 0.037* |  | -0.239 | 0.020* |
| Isthmus cingulate | -0.132 | 0.203 |  | -0.033 | 0.753 |
| Lateral occipital | 0.066 | 0.528 |  | 0.076 | 0.465 |
| Lateral orbitofrontal | -0.138 | 0.183 |  | -0.230 | 0.025* |
| lingual | 0.031 | 0.767 |  | 0.055 | 0.598 |
| Medial orbitofrontal | -0.015 | 0.883 |  | -0.015 | 0.888 |
| Middle temporal | -0.219 | 0.033* |  | -0.214 | 0.037* |
| Parahippocampal | -0.185 | 0.073 |  | -0.196 | 0.057 |
| Paracentral | 0.106 | 0.307 |  | 0.023 | 0.825 |
| Parsopercularis | -0.025 | 0.813 |  | -0.189 | 0.066 |
| Parsorbitalis | -0.136 | 0.187 |  | -0.121 | 0.242 |
| Parstriangularis | 0.016 | 0.876 |  | 0.005 | 0.960 |
| Pericalcarine | -0.089 | 0.390 |  | -0.007 | 0.947 |
| Postcentral | -0.023 | 0.826 |  | -0.011 | 0.919 |
| Posterior cingulate | -0.075 | 0.471 |  | -0.080 | 0.442 |
| Precentral | 0.082 | 0.432 |  | 0.196 | 0.057 |
| Precuneus | -0.052 | 0.616 |  | -0.275 | 0.007** |
| Rostral anterior cingulate | -0.202 | 0.050* |  | -0.077 | 0.458 |
| Rostral middle frontal | -0.135 | 0.193 |  | -0.214 | 0.037* |
| Superior frontal | -0.139 | 0.179 |  | -0.189 | 0.067 |
| Superior parietal | -0.144 | 0.163 |  | -0.094 | 0.363 |
| Superior temporal | -0.145 | 0.160 |  | -0.187 | 0.070 |
| Supramarginal | -0.083 | 0.422 |  | -0.085 | 0.414 |
| Transverse temporal | 0.063 | 0.542 |  | -0.193 | 0.061 |
| Insula | -0.063 | 0.543 |  | -0.227 | 0.027* |

Adjusted for sex, APOE status, cognitive status, age, and education

## **Table S8. Correlation results between pTau217 and brain cortex thickness in Aβ-negative groups.**

|  | Left hemisphere | |  | Right hemisphere | |
| --- | --- | --- | --- | --- | --- |
| Brain region | Correlation | p_value |  | Correlation | p_value |
| Caudal anterior cingulate | 0.124 | 0.159 |  | -0.057 | 0.515 |
| Caudal middle frontal | -0.054 | 0.537 |  | -0.086 | 0.327 |
| Cuneus | -0.141 | 0.108 |  | -0.091 | 0.301 |
| Entorhinal | -0.272 | 0.002** |  | -0.144 | 0.100 |
| Fusiform | -0.147 | 0.093 |  | -0.129 | 0.143 |
| Inferior parietal | -0.099 | 0.259 |  | -0.205 | 0.019* |
| Inferior temporal | -0.142 | 0.105 |  | -0.020 | 0.818 |
| Isthmus cingulate | -0.086 | 0.331 |  | -0.042 | 0.635 |
| Lateral occipital | 0.060 | 0.499 |  | -0.025 | 0.776 |
| Lateral orbitofrontal | -0.058 | 0.513 |  | -0.123 | 0.163 |
| lingual | -0.088 | 0.320 |  | -0.051 | 0.564 |
| Medial orbitofrontal | -0.090 | 0.307 |  | -0.081 | 0.355 |
| Middle temporal | -0.136 | 0.121 |  | -0.169 | 0.053 |
| Parahippocampal | -0.144 | 0.101 |  | -0.131 | 0.136 |
| Paracentral | 0.055 | 0.534 |  | 0.018 | 0.836 |
| Parsopercularis | -0.022 | 0.800 |  | -0.115 | 0.190 |
| Parsorbitalis | -0.210 | 0.016* |  | -0.066 | 0.454 |
| Parstriangularis | -0.078 | 0.378 |  | -0.161 | 0.066 |
| Pericalcarine | -0.127 | 0.150 |  | -0.105 | 0.233 |
| Postcentral | -0.236 | 0.007** |  | -0.086 | 0.327 |
| Posterior cingulate | 0.006 | 0.944 |  | -0.058 | 0.510 |
| Precentral | -0.016 | 0.855 |  | -0.199 | 0.022* |
| Precuneus | -0.164 | 0.062 |  | -0.077 | 0.382 |
| Rostral anterior cingulate | -0.144 | 0.101 |  | -0.192 | 0.028* |
| Rostral middle frontal | -0.085 | 0.332 |  | -0.096 | 0.273 |
| Superior frontal | -0.067 | 0.445 |  | -0.040 | 0.651 |
| Superior parietal | -0.132 | 0.132 |  | -0.214 | 0.014* |
| Superior temporal | -0.092 | 0.297 |  | -0.096 | 0.273 |
| Supramarginal | -0.106 | 0.227 |  | -0.167 | 0.057 |
| Transverse temporal | -0.014 | 0.876 |  | 0.033 | 0.709 |
| Insula | -0.126 | 0.153 |  | -0.015 | 0.866 |

Adjusted for sex, APOE status, cognitive status, age, and education

## **Table S9. Linear regression results for temporal cortex thickness in all participants, Aβ-positive and Aβ-negative groups.** (adjusted for sex, APOE status, cognitive status, age, and education)

| **Group** | **Model** | **Variable** | **Estimate** | **Std.Error** | **t.value** | **p.value** | **p.adjusted** |
| --- | --- | --- | --- | --- | --- | --- | --- |
| All participants | Left  temporal cortex  thickness | (Intercept) | 2.812 | 0.058 | 48.379 | < 0.001 | < 0.001*** |
|  |  | Hcy | -0.005 | 0.006 | -0.715 | 0.475 | 0.792 |
|  |  | Covariates | -0.001 | 0.021 | -0.050 | 0.960 | 0.960 |
|  |  | Hcy:Ptau217 | -0.011 | 0.002 | -5.117 | < 0.001 | < 0.001*** |
|  |  | Hcy:SUVRs | 0.002 | 0.005 | 0.469 | 0.640 | 0.800 |
|  | Right  temporal cortex  thickness | (Intercept) | 2.863 | 0.063 | 45.546 | < 0.001 | < 0.001*** |
|  |  | Hcy | -0.004 | 0.007 | -0.551 | 0.582 | 0.860 |
|  |  | Covariates | -0.009 | 0.022 | -0.402 | 0.688 | 0.860 |
|  |  | Hcy:Ptau217 | -0.013 | 0.002 | -5.606 | < 0.001 | < 0.001*** |
|  |  | Hcy:SUVRs | 0.001 | 0.005 | 0.137 | 0.891 | 0.891 |
| Aβ+  positive | Left  temporal cortex  thickness | (Intercept) | 2.800 | 0.095 | 29.497 | < 0.001 | < 0.001*** |
|  |  | Hcy | -0.011 | 0.010 | -1.065 | 0.290 | 0.362 |
|  |  | Covariates | -0.023 | 0.034 | -0.654 | 0.515 | 0.515 |
|  |  | Hcy:Ptau217 | -0.010 | 0.003 | -3.578 | 0.001 | 0.001** |
|  |  | Hcy:SUVRs | 0.009 | 0.007 | 1.215 | 0.227 | 0.362 |
|  | Right  temporal cortex  thickness | (Intercept) | 2.729 | 0.111 | 24.653 | < 0.001 | < 0.001*** |
|  |  | Hcy | -0.014 | 0.012 | -1.206 | 0.231 | 0.289 |
|  |  | Covariates | 0.008 | 0.040 | 0.188 | 0.851 | 0.851 |
|  |  | Hcy:Ptau217 | -0.012 | 0.003 | -3.591 | 0.001 | 0.001** |
|  |  | Hcy:SUVRs | 0.013 | 0.008 | 1.595 | 0.114 | 0.190 |
| Aβ-positive | Left  temporal cortex  thickness | (Intercept) | 2.782 | 0.074 | 37.653 | < 0.001 | < 0.001*** |
|  |  | Hcy | 0.006 | 0.011 | 0.547 | 0.585 | 0.732 |
|  |  | Covariates | 0.018 | 0.025 | 0.718 | 0.474 | 0.732 |
|  |  | Hcy:Ptau217 | -0.046 | 0.021 | -2.175 | 0.031 | 0.079 |
|  |  | Hcy:SUVRs | -0.003 | 0.009 | -0.287 | 0.775 | 0.775 |
|  | Right  temporal cortex  thickness | (Intercept) | 2.935 | 0.070 | 41.785 | < 0.001 | < 0.001*** |
|  |  | Hcy | 0.004 | 0.011 | 0.419 | 0.676 | 0.676 |
|  |  | Covariates | -0.022 | 0.024 | -0.901 | 0.369 | 0.462 |
|  |  | Hcy:Ptau217 | -0.027 | 0.020 | -1.342 | 0.182 | 0.455 |
|  |  | Hcy:SUVRs | -0.008 | 0.008 | -1.004 | 0.317 | 0.462 |

Abbreviations: Aβ, amyloid β; pTau, phosphorylated tau; tTau, total tau; SUVRs, Standardized Uptake Value Ratios. Significance levels are indicated as *** (p < 0.001), ** (p < 0.01), and * (p < 0.05). p.adjusted means a false discovery rate (FDR) correction was applied.

## **Table S10. Linear regression results for temporal cortex thickness in all participants, Aβ-positive and Aβ-negative groups excluding missing data. (**adjusted for sex, APOE status, cognitive status, age, and education) (N = 220)

| **Group** | **Model** | **Variable** | **Estimate** | **Std.Error** | **t.value** | **p.value** | **p.adjusted** |
| --- | --- | --- | --- | --- | --- | --- | --- |
| All participants | Left  temporal cortex  thickness | (Intercept) | 2.792 | 0.062 | 44.861 | <0.001 | <0.001*** |
|  |  | Hcy | -0.003 | 0.007 | -0.488 | 0.626 | 0.782 |
|  |  | Covariates | 0.004 | 0.022 | 0.192 | 0.848 | 0.848 |
|  |  | Hcy:Ptau217 | -0.012 | 0.002 | -5.055 | <0.001 | <0.001*** |
|  |  | Hcy:SUVRs | 0.003 | 0.005 | 0.506 | 0.613 | 0.782 |
|  | Right  temporal cortex  thickness | (Intercept) | 2.869 | 0.068 | 42.384 | <0.001 | <0.001*** |
|  |  | Hcy | -0.004 | 0.008 | -0.571 | 0.569 | 0.800 |
|  |  | Covariates | -0.011 | 0.024 | -0.469 | 0.640 | 0.800 |
|  |  | Hcy:Ptau217 | -0.014 | 0.002 | -5.430 | <0.001 | <0.001*** |
|  |  | Hcy:SUVRs | 0.001 | 0.006 | 0.223 | 0.824 | 0.824 |
| Aβ+  positive | Left  temporal cortex  thickness | (Intercept) | 2.786 | 0.104 | 26.693 | <0.001 | <0.001*** |
|  |  | Hcy | -0.010 | 0.011 | -0.884 | 0.379 | 0.474 |
|  |  | Covariates | -0.018 | 0.038 | -0.486 | 0.628 | 0.628 |
|  |  | Hcy:Ptau217 | -0.010 | 0.003 | -3.468 | 0.001 | 0.002 |
|  |  | Hcy:SUVRs | 0.009 | 0.008 | 1.118 | 0.267 | 0.445 |
|  | Right  temporal cortex  thickness | (Intercept) | 2.738 | 0.122 | 22.453 | <0.001 | <0.001*** |
|  |  | Hcy | -0.013 | 0.013 | -1.055 | 0.294 | 0.368 |
|  |  | Covariates | 0.003 | 0.044 | 0.057 | 0.955 | 0.955 |
|  |  | Hcy:Ptau217 | -0.012 | 0.003 | -3.564 | 0.001 | 0.001** |
|  |  | Hcy:SUVRs | 0.013 | 0.009 | 1.442 | 0.153 | 0.255 |
| Aβ-positive | Left  temporal cortex  thickness | (Intercept) | 2.763 | 0.078 | 35.272 | <0.001 | <0.001*** |
|  |  | Hcy | 0.005 | 0.013 | 0.384 | 0.702 | 0.877 |
|  |  | Covariates | 0.022 | 0.026 | 0.846 | 0.399 | 0.666 |
|  |  | Hcy:Ptau217 | -0.042 | 0.023 | -1.867 | 0.064 | 0.161 |
|  |  | Hcy:SUVRs | -<0.001 | 0.010 | -0.048 | 0.962 | 0.962 |
|  | Right  temporal cortex  thickness | (Intercept) | 2.938 | 0.076 | 38.755 | <0.001 | <0.001*** |
|  |  | Hcy | 0.003 | 0.013 | 0.270 | 0.788 | 0.788 |
|  |  | Covariates | -0.022 | 0.025 | -0.849 | 0.397 | 0.535 |
|  |  | Hcy:Ptau217 | -0.026 | 0.022 | -1.168 | 0.245 | 0.535 |
|  |  | Hcy:SUVRs | -0.008 | 0.010 | -0.796 | 0.428 | 0.535 |

Abbreviations: Aβ, amyloid β; pTau, phosphorylated tau; tTau, total tau; SUVRs, Standardized Uptake Value Ratios. Significance levels are indicated as *** (p < 0.001), ** (p < 0.01), and * (p < 0.05). p.adjusted means a false discovery rate (FDR) correction was applied.

## **Table S11. Linear regression results for temporal cortex thickness in all participants, Aβ-positive and Aβ-negative groups with males. (**adjusted for APOE status, cognitive status, age, and education) (N = 70)

| **Group** | **Model** | **Variable** | **Estimate** | **Std.Error** | **t.value** | **p.value** | **p.adjusted** |
| --- | --- | --- | --- | --- | --- | --- | --- |
| All participants | Left  temporal cortex  thickness | (Intercept) | 3.116 | 0.201 | 15.528 | <0.001 | <0.001*** |
|  |  | Hcy | -0.009 | 0.013 | -0.639 | 0.525 | 0.525 |
|  |  | Covariates | -0.006 | 0.003 | -2.475 | 0.016 | 0.027* |
|  |  | Hcy:Ptau217 | -0.018 | 0.005 | -3.579 | 0.001 | 0.002* |
|  |  | Hcy:SUVRs | 0.017 | 0.010 | 1.642 | 0.105 | 0.132 |
|  | Right  temporal cortex  thickness | (Intercept) | 3.253 | 0.191 | 17.003 | <0.001 | <0.001*** |
|  |  | Hcy | -0.002 | 0.013 | -0.164 | 0.870 | 0.870 |
|  |  | Covariates | -0.007 | 0.002 | -2.706 | 0.009 | 0.014* |
|  |  | Hcy:Ptau217 | -0.022 | 0.005 | -4.635 | <0.001 | <0.001*** |
|  |  | Hcy:SUVRs | 0.006 | 0.010 | 0.577 | 0.566 | 0.707 |
| Aβ+  positive | Left  temporal cortex  thickness | (Intercept) | 3.094 | 0.325 | 9.507 | <0.001 | <0.001*** |
|  |  | Hcy | -0.010 | 0.021 | -0.485 | 0.631 | 0.631 |
|  |  | Covariates | -0.006 | 0.004 | -1.507 | 0.144 | 0.240 |
|  |  | Hcy:Ptau217 | -0.015 | 0.006 | -2.428 | 0.022 | 0.056 |
|  |  | Hcy:SUVRs | 0.015 | 0.013 | 1.148 | 0.261 | 0.327 |
|  | Right  temporal cortex  thickness | (Intercept) | 3.339 | 0.372 | 8.979 | <0.001 | <0.001*** |
|  |  | Hcy | -0.004 | 0.024 | -0.189 | 0.852 | 0.852 |
|  |  | Covariates | -0.010 | 0.004 | -2.222 | 0.035 | 0.059 |
|  |  | Hcy:Ptau217 | -0.023 | 0.007 | -3.326 | 0.003 | 0.007* |
|  |  | Hcy:SUVRs | 0.015 | 0.015 | 1.015 | 0.320 | 0.399 |
| Aβ-positive | Left  temporal cortex  thickness | (Intercept) | 3.033 | 0.264 | 11.482 | <0.001 | <0.001*** |
|  |  | Hcy | 0.016 | 0.029 | 0.544 | 0.590 | 0.590 |
|  |  | Covariates | -0.007 | 0.003 | -1.979 | 0.056 | 0.093 |
|  |  | Hcy:Ptau217 | -0.109 | 0.044 | -2.475 | 0.018 | 0.046* |
|  |  | Hcy:SUVRs | 0.017 | 0.023 | 0.755 | 0.455 | 0.569 |
|  | Right  temporal cortex  thickness | (Intercept) | 3.129 | 0.201 | 15.569 | <0.001 | <0.001*** |
|  |  | Hcy | 0.013 | 0.022 | 0.607 | 0.548 | 0.548 |
|  |  | Covariates | -0.004 | 0.003 | -1.345 | 0.188 | 0.469 |
|  |  | Hcy:Ptau217 | -0.030 | 0.034 | -0.885 | 0.382 | 0.514 |
|  |  | Hcy:SUVRs | -0.014 | 0.017 | -0.832 | 0.411 | 0.514 |

Abbreviations: Aβ, amyloid β; pTau, phosphorylated tau; tTau, total tau; SUVRs, Standardized Uptake Value Ratios. Significance levels are indicated as *** (p < 0.001), ** (p < 0.01), and * (p < 0.05). p.adjusted means a false discovery rate (FDR) correction was applied.

## **Table S12. Linear regression results for temporal cortex thickness in all participants, Aβ-positive and Aβ-negative groups with females.** (adjusted for APOE status, cognitive status, age, and education) (N = 166)

| **Group** | **Model** | **Variable** | **Estimate** | **Std.Error** | **t.value** | **p.value** | **p.adjusted** |
| --- | --- | --- | --- | --- | --- | --- | --- |
| All participants | Left  temporal cortex  thickness | (Intercept) | 3.003 | 0.101 | 29.684 | <0.001 | <0.001*** |
|  |  | Hcy | -0.003 | 0.007 | -0.366 | 0.715 | 0.835 |
|  |  | Covariates | -0.003 | 0.001 | -1.707 | 0.090 | 0.149 |
|  |  | Hcy:Ptau217 | -0.011 | 0.002 | -4.520 | <0.001 | <0.001*** |
|  |  | Hcy:SUVRs | -0.001 | 0.005 | -0.208 | 0.835 | 0.835 |
|  | Right  temporal cortex  thickness | (Intercept) | 3.261 | 0.113 | 28.880 | <0.001 | <0.001*** |
|  |  | Hcy | -0.006 | 0.008 | -0.791 | 0.430 | 0.509 |
|  |  | Covariates | -0.006 | 0.002 | -3.804 | <0.001 | <0.001*** |
|  |  | Hcy:Ptau217 | -0.013 | 0.003 | -4.905 | <0.001 | <0.001*** |
|  |  | Hcy:SUVRs | 0.004 | 0.006 | 0.661 | 0.509 | 0.509 |
| Aβ+  positive | Left  temporal cortex  thickness | (Intercept) | 2.731 | 0.197 | 13.832 | <0.001 | <0.001*** |
|  |  | Hcy | -0.013 | 0.013 | -1.021 | 0.311 | 0.487 |
|  |  | Covariates | 0.001 | 0.003 | 0.272 | 0.786 | 0.786 |
|  |  | Hcy:Ptau217 | -0.009 | 0.003 | -2.595 | 0.012 | 0.029* |
|  |  | Hcy:SUVRs | 0.008 | 0.009 | 0.866 | 0.390 | 0.487 |
|  | Right  temporal cortex  thickness | (Intercept) | 2.958 | 0.223 | 13.294 | <0.001 | <0.001*** |
|  |  | Hcy | -0.020 | 0.014 | -1.407 | 0.164 | 0.205 |
|  |  | Covariates | -0.003 | 0.003 | -0.901 | 0.371 | 0.371 |
|  |  | Hcy:Ptau217 | -0.010 | 0.004 | -2.628 | 0.011 | 0.027* |
|  |  | Hcy:SUVRs | 0.016 | 0.010 | 1.549 | 0.126 | 0.205 |
| Aβ-positive | Left  temporal cortex  thickness | (Intercept) | 3.127 | 0.115 | 27.109 | <0.001 | <0.001*** |
|  |  | Hcy | -0.001 | 0.011 | -0.079 | 0.937 | 0.937 |
|  |  | Covariates | -0.004 | 0.002 | -2.412 | 0.018 | 0.045* |
|  |  | Hcy:Ptau217 | -0.012 | 0.022 | -0.553 | 0.582 | 0.850 |
|  |  | Hcy:SUVRs | -0.004 | 0.009 | -0.413 | 0.680 | 0.850 |
|  | Right  temporal cortex  thickness | (Intercept) | 3.372 | 0.125 | 26.996 | <0.001 | <0.001*** |
|  |  | Hcy | -0.006 | 0.012 | -0.519 | 0.605 | 0.738 |
|  |  | Covariates | -0.007 | 0.002 | -4.108 | <0.001 | <0.001*** |
|  |  | Hcy:Ptau217 | -0.018 | 0.024 | -0.757 | 0.451 | 0.738 |
|  |  | Hcy:SUVRs | 0.003 | 0.009 | 0.335 | 0.738 | 0.738 |

Abbreviations: Aβ, amyloid β; pTau, phosphorylated tau; tTau, total tau; SUVRs, Standardized Uptake Value Ratios. Significance levels are indicated as *** (p < 0.001), ** (p < 0.01), and * (p < 0.05). p.adjusted means a false discovery rate (FDR) correction was applied.
